# Design and Microwave Assisted Synthesis of Coumarin Derivatives as PDE Inhibitors

**DOI:** 10.1155/2016/9890630

**Published:** 2016-02-21

**Authors:** Mahadev N. Kumbar, Ravindra R. Kamble, Atulkumar A. Kamble, Sujith Raj Salian, Sandhya Kumari, Ramya Nair, Guruprasad Kalthur, Satish Kumar Adiga, D. Jagadeesh Prasad

**Affiliations:** ^1^Department of Chemistry, Karnatak University, Pavate Nagar, Dharwad 580003, India; ^2^Centre of Excellence in Clinical Embryology, Level 2, Central Research Lab, Kasturba Medical College, Manipal University, Manipal, Karnataka 576104, India; ^3^Department of Studies in Chemistry, Mangalore University, Konaje, Karnataka 574199, India

## Abstract

Coumarins appended to benzimidazole through pyrazole are designed and synthesized using microwave irradiation. These compounds were analyzed for phosphodiesterase (PDE) inhibition indirectly by motility pattern in human spermatozoa. Some of the synthesized compounds, namely,** 5d**,** 5e**,** 5f**,** 5g**,** 5h**, and** 5k,** have exhibited potent inhibitory activity on PDE.

## 1. Introduction

The development of simple, mild, practicable, cheap eco-benign method for the synthesis of heterocycles has grabbed the attention of researchers. In particular, microwave assisted organic synthesis has become a rapidly growing field in organic chemistry as this technique makes reaction time shorter and tolerates wide range reactions which are best suited to the increased demands of industry [1] and some of the synthesized compounds were screened by PDE.

PDE inhibitors are therapeutic agents which target PDE isoenzymes and inhibit the catabolism of the secondary messengers such as cyclic adenosine monophosphate (cAMP) and cyclic guanosine monophosphate (cGMP), thus prolonging the biological effect determined by the type of cell involved. Cyclic nucleotide phosphodiesterases (PDEs) catalyze the hydrolysis of cAMP and/or cGMP. They function in conjunction with* adenylyl* and* guanylyl cyclases* to regulate the amplitude and duration of cell signaling mechanisms mediated* via* cAMP and cGMP. They therefore serve to regulate a range of biological responses to first messengers such as light vascular resistance, cardiac output, visceral motility, immune response [2], inflammation [3], neuroplasticity, vision [4], and reproduction [5]. Phosphodiesterases (PDEs) modulate the activity of cyclic nucleotides by regulating their degradation. PDEs are critical determinants for modulation of cellular levels of cAMP and/or cGMP by many stimuli [6]. Thus, the ubiquitously present PDEs play a pivotal role in regulating cell signaling the breakdown of cAMP and cGMP.

The fold selectivity for PDE5 over PDE11A4 for Sildenafil (1000-fold selectivity) and Vardenafil (9000-fold selectivity) reveals that these drugs are very unlikely to cross-react with PDE11A4 in patients treated with these medications. On the contrary, it was suggested to use the newest PDE5 inhibitor Tadalafil with caution. In fact, the 40-fold selectivity ratio of Tadalafil for PDE5 over PDE11A4 is significantly lower than those reported with the other two drugs and is nearly the same as that reported for Sildenafil over PDE1 (41-fold). In this case, it has been suggested that the 41-fold selectivity of Sildenafil for PDE5 over PDE1 may induce vasodilatation, flushing, and tachycardia. Within human PDE11A family, the alternative splicing leads to generation of proteins which display unique properties [7, 8].

**Figure 1:**
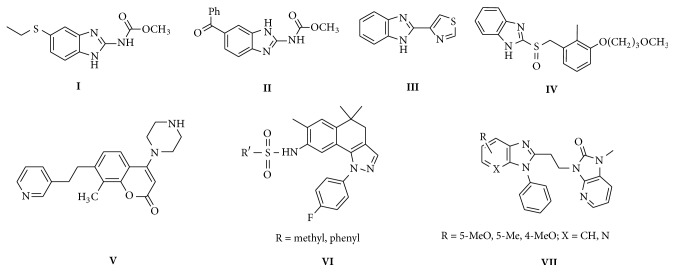
Some of the benzimidazole drugs and known PDE inhibitors.

Benzimidazole nucleus is the key building block for numerous drugs that play beneficial roles in the functioning of biologically important molecules. Specifically, this nucleus is a constituent of Vit B_12_ and many currently existing medications. Almost all benzimidazoles with different heterocyclic substituents led to essential modification in their physicochemical, metabolic, and pharmacokinetic properties [9]. Benzimidazole is a core structural moiety found in some of the important drugs like albendazole (**I**), mebendazole (**II**), thiabendazole (**III**), rabeprazole (**IV**), and so forth. Literature survey revealed that coumarin (**V**) scaffolds were proved to increase the cAMP levels through the specific inhibition of PDE3 in accordance with their common structural features [10] and pyrazole (**VI**) derivatives have been explored for the identification of phosphodiesterase (PDE4) inhibitors as is exemplified by the discovery and development of tofimilast [11]; also most of the benzimidazole (**VII**) derivatives have shown very prominent PDE (10A) activity [12]. Coumarin, pyrazole, and benzimidazole core structural moiety are found in some of the important PDE inhibition compounds by Yang et al. [13] (Figure 1).

Also, the scaffolds containing coumarin, pyrazole and benzimidazole are the key moieties in heterocyclic chemistry and are important structural units of various natural and synthetic biologically active molecules. They are known to possess a wide range of pharmacological activities that include antimicrobial and anti-inflammatory activities. Many coumarin derivatives have shown anticancer, anticoagulant, anti-inflammatory, antimicrobial, antioxidant, antiviral, and cardiovascular activities [14–22].

**Figure 2:**
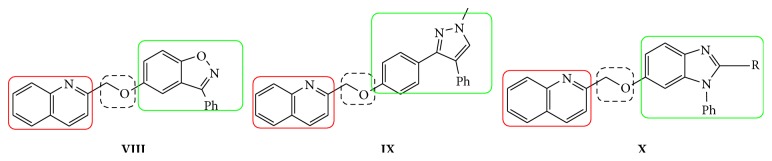
Representative drug molecules as PDE inhibitors** VIII**–**X**.

**Figure 3:**
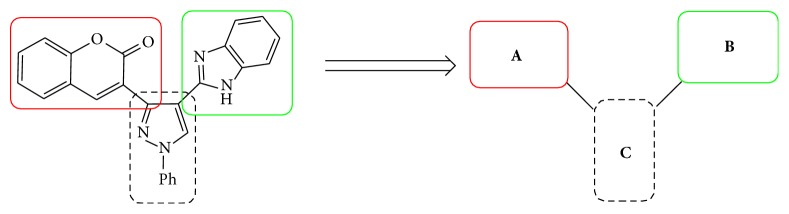
Designed 3-(4-(*1H*-benzo[*d*]imidazol-2-yl)-1-phenyl-*1H*-pyrazol-3-yl)-*2H*-chromen-2-ones derivatives** 5a**–**x**.

During drug development of PDEs, it was believed that SAR of pyrazole and pyrazole replacements that remove the hydrogen bond donor were very promising. The structural analogs viz., compounds** VIII** and** X** contain benzoxazole and benzimidazole attached to quinoline through oxygen as linker group. These have shown possess promising PDE inhibition as reported by Hamaguchi et al [23]. Also substitution of the pyrazole with simple alkyl groups retained potency while adding minimal molecular weight. The methyl substituted pyrazole** IX** (Figure 2) in an* in vitro* P-glycoprotein (PgP) overexpressing cell line had improved the efflux ratios. These above drug development results and the structures of various classes of clinically established PDEs conform to a broadly accepted pharmacophore (Figure 3). This suggests that three important structural requirements are to be present to show PDE inhibition. Those three important parameters are viz., (i) fused heterocyclic ring for good oral bioavailability (ring** A**)and (ii) a five-membered heterocycle or alkyl chain (**B**) which is connected to another fused five-membered heterocycle (ring** C**) [24].

In view of the above and in search of biologically potent novel heterocyclic scaffolds, herein we report facile and inexpensive method for the synthesis of coumarin with pyrazole nucleus and functionalized benzimidazoles in a single moiety with an expectation to obtain potent PDE inhibitor.

## 2. Results and Discussion

### 2.1. Chemistry

**Scheme 1:**
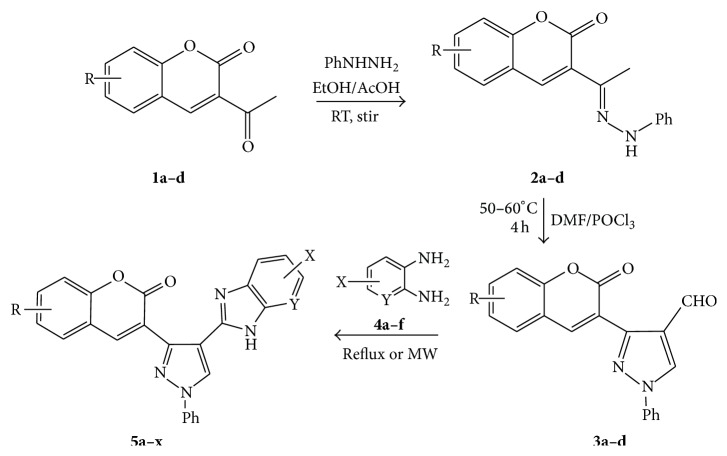
Synthetic route for title compounds** 5a**–**x**, where** 5a**; R = -H, X = -H, Y = H,** 5b**; R = -H, X = 6-Cl, Y = H,** 5c**; R = -H, X = 6-CH_3_, Y = H,** 5d**; R = -H, X = 6-NO_2_, Y = H,** 5e**; R = -H, X = 6-Br, Y = N,** 5f**; R = -H, X = 5,7-dimethyl, Y = H,** 5g**; R = -6Cl, X = -H, Y = H,** 5h**; R = -6Cl, X = -4Cl, Y = H,** 5i**; R = -6Cl, X = -CH_3_, Y = H,** 5j**; R = -6Cl, X = 6-NO_2_, Y = H,** 5k**; R = -6Cl, X = 6-Br, Y = N,** 5l**; R = -6Cl, X = 5,7-dimethyl, Y = H,** 5m**; R = -6Br, X = -H, Y = H,** 5n**; R = -6Br, X = -4Cl, Y = H,** 5o**; R = -6Br, X = -CH_3_, Y = H,** 5p**; R = -6Br, X = 6-NO_2_, Y = H,** 5q**; R = -6Br, X = 5-Br, Y = N,** 5r**; R = -6Br, X = 5,7-dimethyl, Y = H,** 5s**; R = 8-OCH_3_, X = -H, Y = H,** 5t**; R = 8-OCH_3_, X = 4-Cl, Y = H,** 5u**; R = 8-OCH_3_, X = 6-CH_3_, Y = H,** 5v**; R = 8-OCH_3_, X = 6-NO_2_, Y = H,** 5w**; R = 8-OCH_3_, X = 5-Br, Y = N,** 5x**; R = 8-OCH_3_, X = 5,7-dimethyl, Y = H.

The starting materials 3-acetyl-2*H*-chromen-2-one** 1a**–**d** and 3-(2-oxo-2*H*-chromen-3-yl)-1-phenyl-1*H*-pyrazole-4-carbaldehyde** 3a**–**d** were obtained using literature methods [25]. The synthetic protocols for the title compounds* 3-(4-(1H-benzo[d]imidazol-2-yl)-1-phenyl-1H-pyrazol-3-yl)-2H-chromen-2-ones* **5a**–**x** are outlined in Scheme 1. Also an optimum condition was established under microwave irradiation for the synthesis of the title compounds** 5a**–**x** by condensation of* o*-arylenediamines **4a**–**f** and 3-(2-oxo-2*H*-chromen-3-yl)-1-phenyl-1*H*-pyrazole-4-carbaldehydes** 3a**–**d** in ethanol in 5–8 min with excellent yields. Compared to conventional method (4–6 hrs), microwave irradiation greatly reduced the reaction time from 3-4 h to 6–8 min. The yield of the product was also increased up to 94% (Table 1).

Structures of all the synthesized compounds** 5a–x** were confirmed by various spectroscopic techniques, viz., IR, ^1^H, ^13^C NMR, MS, and elemental analyses. The compounds have shown strong adsorption band for carbonyl of coumarin and N-H of benzimidazole ring at 1711–1737 and 3345–3368 cm^−1^ respectively. In case of ^1^H NMR spectra, all the compounds exhibited a singlet in the range 10.42–13.36 ppm. for benzimidazole N-H ring and coumarin C_4_H at 7.69–7.92 ppm. The aromatic protons of all pyrazole rings appeared as multiplets in the range 7.32–7.98 ppm. In case of ^13^C NMR spectral study, the numbers of signals are consistent with number of magnetically nonequivalent carbon atoms in the molecule and in mass spectra all the synthesized title compounds have shown the molecular ion peaks at their respective* m/z* values.

## 3. PDE Inhibition Study

### 3.1. Effect on Motility of Frozen-Thawed Human Spermatozoa

To assess the effect of title compounds on the motility pattern, human spermatozoa subjected to freeze-thaw process was used. It is well documented that freeze-thaw process can have detrimental effect on the motility and survival of the spermatozoa [26]. Therefore, they can be an excellent model to study the effect of any test compounds on the motility [27].

In the present study, we assessed the motility pattern in spermatozoa processed with media containing various compounds manually at 1, 4, and 24 h after incubation* in vitro*. Three arbitrary doses were selected (0.5, 1.0, and 5.0 *µ*g/mL) for each compound to assess their effect on sperm motility. Pentoxifylline (PTF), which is known to enhance the human sperm motility by elevating intracellular cyclic adenosine monophosphate (cAMP) level [27], was taken as a positive control. Based on the previous report, PTF at 1 mM concentration was used [28]. Since the test compounds were not soluble in water, dimethyl sulfoxide (DMSO, 0.05%) was used as vehicle control.

The sperm motility enhancement property of the compounds was categorized as below:

(a) Marginal: which resulted in <5% increase in the motility; (b) Good: which resulted in 5–10% increase in motility; (c) Excellent: which resulted in >10% increase in motility; (d) Poor: where there was no enhancement in motility.

At 1 h after the* in vitro* incubation, the majority of the compounds had marginal effect on the sperm motility compared to control. At this interval, PTF had higher percentage of total and progressively motile spermatozoa compared to all the test compounds. A large number of studies have shown that PTF can trigger the motility in fresh or frozen-thawed spermatozoa as early as 1 h [29], even though the majority of the studies indicate that this effect is mainly due to the elevated cAMP level in the spermatozoa [30], it can also be mediated through tyrosine phosphorylation [31].

Compounds** 5a**,** 5b**,** 5c**,** 5d**,** 5e**,** 5g**,** 5h**, and** 5k** induced marginal enhancement in total motility while compounds** 5a**,** 5b**,** 5c**, and** 5j** resulted in similar effect on progressive motility (Tables 2(a) and 2(b)). A 5–10% increase in total motility was induced by compound** 5j**. However, with respect to progressive motility, compounds** 5d**,** 5e**,** 5f**, and** 5k** came under compounds with good effect on motility. More than 10% increase in progressive motility was observed only for compound** 5g** (at 0.5 *µ*g/mL concentration) and compound** 5h** (at 5 *µ*g/mL concentration).

A time-dependent decrease in total and progressive motility was observed in all the groups. However, the decline was more evident at 24 h interval. This phenomenon has been documented by earlier studies [28]. These changes may be related to the oxidative stress induced by* in vitro* culture conditions. Spermatozoa are highly susceptible to reactive oxygen species (ROS) due to high polyunsaturated fatty acid content in their plasma membrane and negligible amount of cytoplasmic antioxidants.

At 4 h interval, compounds** 5e**,** 5f**, and** 5g** enhanced the progressive motility of spermatozoa by 5–10% while compound** 5h** with two chloro substituents on coumarin and benzimidazole resulted in almost 15% increase in the progressive motility. However, the effect was lower than that of pentoxifylline. The rest of the compounds (**5a**,** 5b**,** 5c**,** 5f**, and** 5j**) induced a marginal increase in motility except in compound** 5e** in which the motility was lower than the control group.

At 24 h interval, the majority of the compounds** (5a**,** 5b**,** 5d**,** 5f**,** 5h**, and** 5j)** had marginally higher percentage of motility or poor motility compounds** (5c**,** 5e**, and** 5g)** compared to control. Interestingly, compound** 5k** with chloro substituent on coumarin and bromo substitution on benzimidazole had higher percentage of motile spermatozoa compared to control (14% higher) and even PTF (9% higher). This indicates that compound** 5k** can help in prolonging the* in vitro* survival of spermatozoa which has significant beneficial role in assisted reproductive technologies such as* in vitro* fertilization. Previous studies have shown that even though PTF has triggering effect on the motility, it induces premature acrosome reaction and drastic decrease in motility at later intervals. In this context, the compounds which increase the motility and longevity of spermatozoa under* in vitro* conditions are of clinical relevance.

Based on the manual motility assessment, we observed that compounds** 5d**,** 5f**,** 5g**,** 5h**, and** 5k** have considerable beneficial role in sperm motility. Therefore, these compounds were further assessed for their effect on the kinematics of spermatozoa at 1 h. However, there was no significant difference in the kinematics of spermatozoa compared to control and PTF (Table 3). In conclusion, the newly synthesized coumarin derivatives have sperm motility enhancing property. However, in this preliminary screening we have taken three arbitrary doses to assess their effect on motility. Compounds** 5g**,** 5h**, and** 5k** show considerable beneficial effect which can be of clinical significance where sperm motility enhancement is achieved under* in vitro* conditions. Compounds** 5g** and** 5h** triggered the motility at early intervals, which was similar to the PTF, while compound** 5k** improved the longevity of the spermatozoa. Since the coumarin is known to have PDE inhibitory function, the motility enhancement could be due to the elevated cAMP level in sperm. However, further detailed studies are essential to understand the mechanism of action of these compounds and their clinical utility.

## 4. Experimental

All the chemicals and reagents were purchased from the Merck and Aldrich chemical suppliers. Melting points (mp) were determined in open capillaries and are uncorrected. The IR spectra were recorded on a Nicolet Impact 410 FT IR spectrometer using KBr pellets (range 4000–500 cm^−1^). The ^1^H NMR spectra were recorded at 400 MHz on Bruker Avance FT NMR spectrometer in DMSO-d_6_ solvent with TMS as internal standard. ^13^C NMR spectra were recorded at 100 MHz on Bruker Avance FT NMR spectrometer in DMSO-d_6_ solvent with TMS as internal standard. The mass spectra were recorded on Shimadzu GC-MS operating at 70 eV. Thin-layer chromatography (TLC) was performed on 0.20 mm Aluchrosep silica gel 60 F254 plates (SD Fine, Mumbai). Microwave irradiation experiments were carried out using CEM Discover SP Microwave Synthesizer equipped with IR sensor to monitor reaction temperatures.

## 5. General Procedures for the Preparation of Title Compounds 5a–x

3-(2-Oxo-2H-chromen-3-yl)-1-phenyl-1H-pyrazole-4-carbaldehydes (**3a**–**d**, 1 mmol) were prepared from 3-acetylcoumarin (**1a**–**d**, 1 mmol), phenylhydrazine (1 mmol), and the intermediate (Schiff base) (**2a**–**d**, 1 mmol) followed by reaction with POCl_3_ in DMF by Vilsmeier Haack formylation strategies [25]. The target compounds** 5a**–**x** were then achieved by stirring substituted carbaldehyde (**3a**–**d**, 1 mmol) with different* o*-arylenediamine (**4a**–**f**, 1 mmol), under reflux for 4-5 hrs. After completion (TLC, hexane : ethylacetate, 7 : 3) of the reaction, the solid obtained was filtered, washed with small quantity of ethanol, and dried. Further, it was recrystallized in methanol to get the pure compound (**5a**–**x**).

### 5.1. Microwave Assisted Procedure for the Preparation of Compounds** 5a–x**

A mixture of 3-(2-oxo-2H-chromen-3-yl)-1-phenyl-1*H*-pyrazole-4-carbaldehyde (**3a**–**d**, 1 mmol) and o-phenylenediamine (**4a**–**f**, 1 mmol) taken in ethanol (5 mL) was introduced into a CEM microwave reaction vessel equipped with magnetic stirrer. The reaction mixture was prestirred for 1 min at room temperature and irradiated at 180 W (150°C) for about 6–8 min (TLC using hexane : ethyl acetate (7 : 3 drops) as eluent). The reaction mixture was then quenched into crushed ice and the crude product was filtered, washed, and dried. Recrystallization from methanol gave pure crystals of the compounds** 5a**–**x**.

(Spectral as well as elemental analyses data are provided as electronic supplementary file in Supplementary Material available online at http://dx.doi.org/10.1155/2016/9890630.)

## 6. Assessment of Phosphodiesterase Inhibition Using Human Spermatozoa as Model

Earlier studies have shown that PDE inhibitors can enhance the sperm motility [32, 33]. Based on this, in the present study we have used an indirect approach to assess the inhibitory effect of the coumarin derivatives on PDE by assessing sperm motility pattern.

### 6.1. Preparation of Test Solution

Stock solution of the test compounds (1 mg/mL) was prepared by dissolving them in dimethyl sulfoxide (DMSO, Sigma, Cat. number D5879). The stock solution was then diluted with Earl's Balanced Salt Solution (EBSS) supplemented with 0.1% bovine serum albumin (BSA) in a ratio of 1 : 200, 1 : 1000, and 1 : 2000 to get working solutions of 5, 1, and 0.5 *µ*g/mL concentrations. The working solutions were preincubated at 37°C and 5% CO_2_, prior to use.

### 6.2. Study Subjects

Infertile men who attended Andrology Laboratory, Kasturba Medical College, Manipal University, Manipal, during the period of June-July 2015 for routine semen evaluation were included in the study. The subjects with a sexual abstinence of 3–5 days were asked to provide the semen samples by masturbation in a sterile container. Following liquefaction, the semen parameters were assessed as described by Kotdawala et al. [34]. The study was approved by the Institutional Ethics Committee of Kasturba Hospital, Kasturba Medical College, Manipal University (IEC. number 155/2015).

### 6.3. Cryopreservation and Thawing

The liquefied semen samples were cryopreserved by rapid freezing method as described previously [34] with minor modifications. Briefly, the semen samples were mixed with equal volume of freezing medium (Sperm Freeze, FertiPro, Cat. number 0344) in a cryovial (Thermoscientific, Nunc, Cat. number 138627) and kept at 4°C for 10 min, in liquid nitrogen (LN2) vapor phase for 5 min, and then finally plunged into liquid nitrogen. The samples were thawed after 1 week by rapid thawing method by placing them at 37°C for 5 min. The cryoprotectant medium was completely removed by mixing the sperm suspension with EBSS medium and centrifuging at 1000 rpm for 8 min. The resultant pellet was resuspended with fresh EBSS medium and used for further analysis.

### 6.4. Sample Preparation

To screen the compounds with enhancing effect on the motility of spermatozoa under* in vitro* conditions, the frozen-thawed semen samples were washed by mixing with equal volume of preincubated Earl's Balanced Salt Solution (EBSS, Sigma Cat. number E2888) followed by centrifugation at 1800 rpm for 8 min. The pellet was then gently mixed with 1 mL of EBSS media containing 0.1% bovine serum albumin (BSA, Sigma, Cat. number A3311) and equally divided into control, vehicle control, and test groups (with average sperm density of 5 × 10^4^ spermatozoa in each group). The motile sperm were extracted by swim up method as described earlier [35]. The sperm suspension was centrifuged at 1200 rpm for 8 min and the resultant pellet was overlaid with EBSS medium containing various test compounds. The sperm suspensions were incubated for 1 h at 37°C and 5% CO_2_ after which the motile sperm were collected from the supernatant.

### 6.5. Motility Assessment

Spermatozoa in the supernatant fraction were assessed for their motility patterns at 1, 4, and 24 h after incubation under light microscope as described earlier [27]. Sperm suspension was (10 *µ*L droplet) placed on a clean microscope slide and a coverslip was placed over it. Spermatozoa with progressive motility and nonprogressive motility and immotile spermatozoa were scored separately from a total of 200 spermatozoa in random fields at 400x magnification.

### 6.6. Sperm Kinematics Using Computer Assisted Semen Analysis (CASA)

The motion characteristics in the spermatozoa were assessed at 1, 4, and 24 h after incubation using CASA system (ISAS, Spain). Briefly, 5 *µ*L of sperm suspension was placed on a clean microscope slide prewarmed at 37°C. The sperm suspension was covered with prewarmed cover slip (22 × 22 mm) and observed under microscope (negative phase contrast, 10x objectives, Proiser, Spain). For each sample, the kinematics was assessed randomly at ten different fields. The parameters such as curvilinear velocity (VCL), straight line velocity (VSL), average path velocity (VAP), amplitude of lateral head displacement (ALH), linearity (LIN), straightness (STR), wobble (WOB), and beat cross frequency (BCF) were analyzed.

## 7. Conclusions

In conclusion, we have reported efficient and environmentally benign methodologies for the synthesis of 3-[4-(1*H*-benzo[d]imidazol-2-yl)-1-phenyl-*1H*-pyrazol-3-yl]-2H-chromen-2-one derivatives** 5a**–**x** by using thermal and by microwave irradiation under neat conditions in presence of ethanol. The reactions carried out under microwave irradiation afforded benzimidazoles in short period of time with excellent yields. Hence, this methodology would make an interesting strategy for the synthesis of various substituted coumarin pyrazole benzimidazoles. Also, compounds** 5g**,** 5h**, and** 5k** exhibited impressive sperm motility enhancing property whereas** 5k** has spermatozoa longevity property.

## Supplementary Material

The supplementary material available online contains spectral characterization of title compounds 5a-x, copies of IR, 1H-NMR, 13C-NMR, and Mass spectra of compounds 5a, 5d and 5e.

## Figures and Tables

**Table 1:** Comparison of conventional and microwave synthesis of title compounds **5a**–**x** from **3a**–**d** and **4a**–**f** in absolute ethanol under optimized conditions.

**3a**–**d**	**4a**–**f**	**5a**–**x**	Conventional		Microwave	
			Time (hr)	Yield (%)	Time (min)	Yield
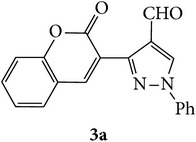	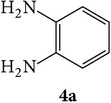	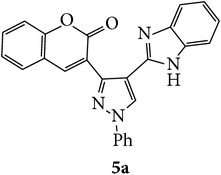	4	39	5	94
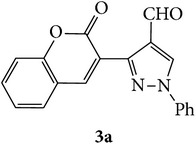	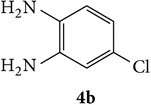	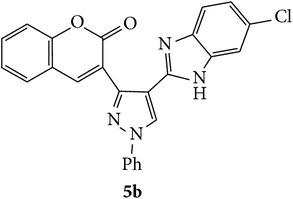	4	35	5	90
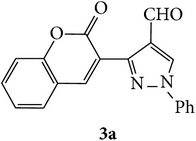	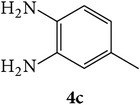	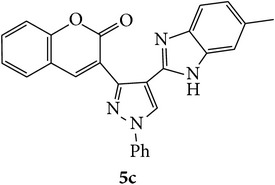	4	38	5	88
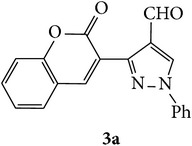	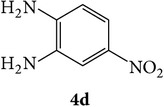	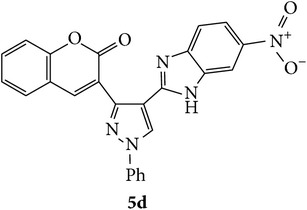	4	32	5	86
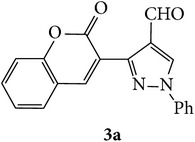	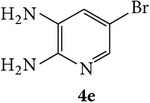	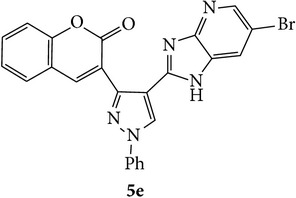	4	34	5	92
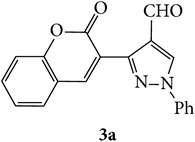	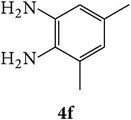	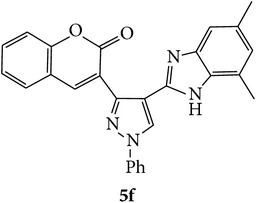	4	35	5	90
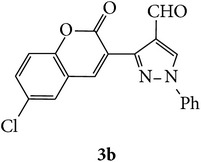	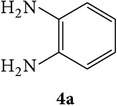	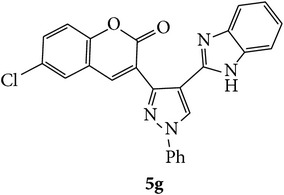	5	40	6	94
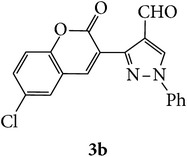	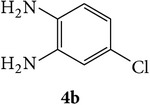	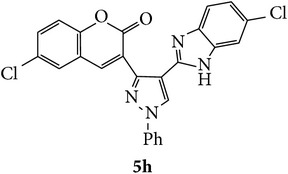	5	32	6	85
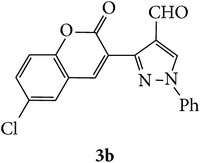	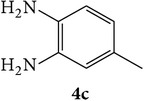	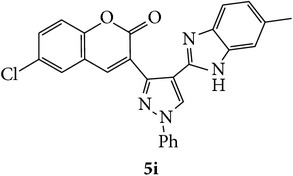	5	30	6	87
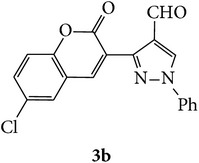	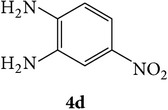	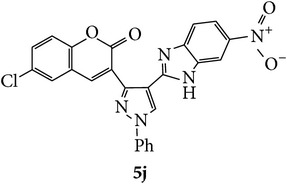	5	35	6	84
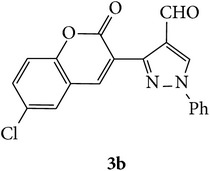	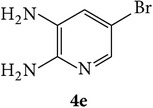	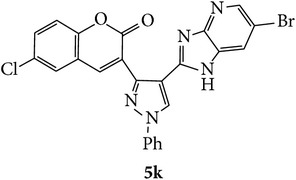	5	34	6	86
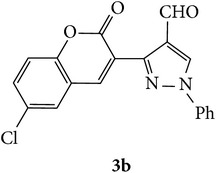	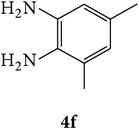	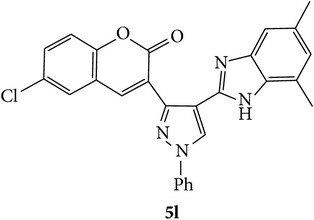	5	37	6	90
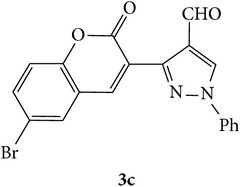	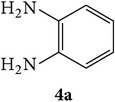	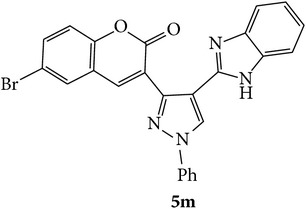	6	33	7	89
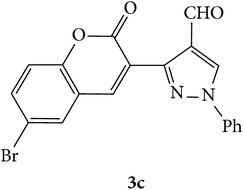	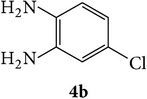	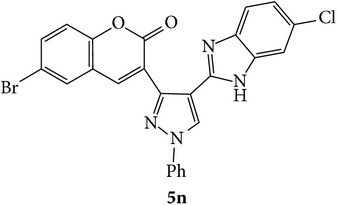	6	36	7	92
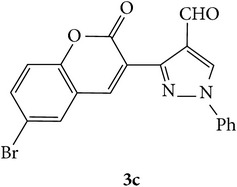	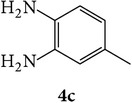	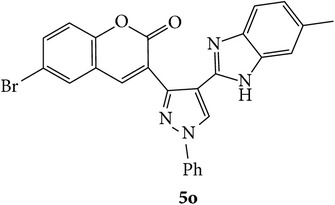	6	39	7	94
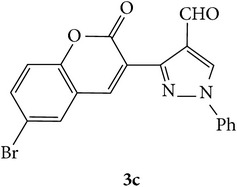	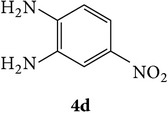	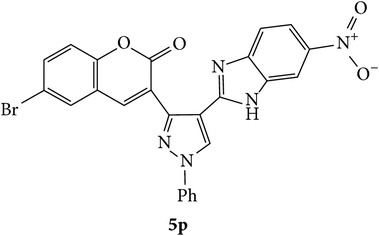	6	36	7	84
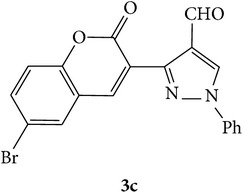	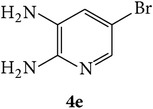	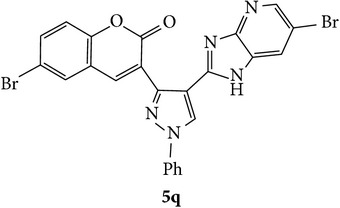	6	31	7	90
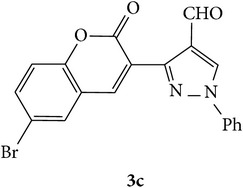	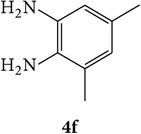	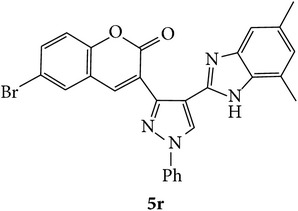	6	34	7	92
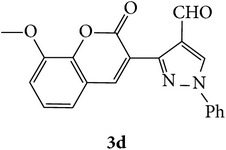	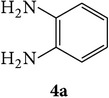	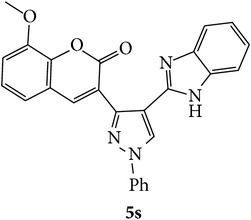	5	38	8	87
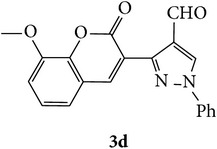	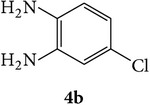	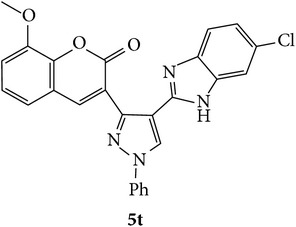	5	40	8	85
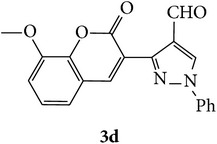	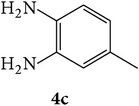	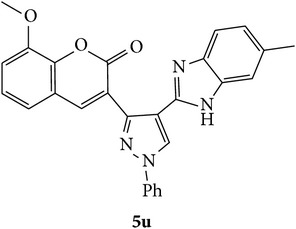	5	32	8	88
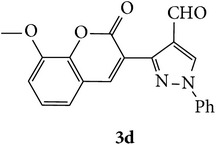	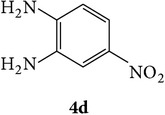	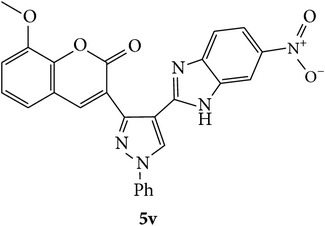	5	36	8	86
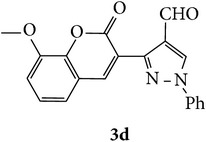	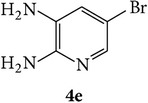	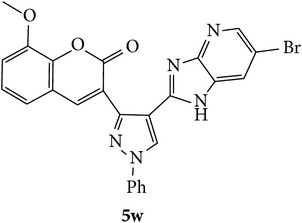	5	38	8	80
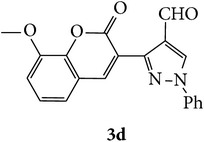	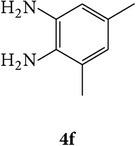	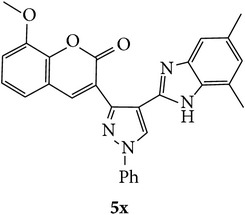	5	34	8	82

**(a):** 

Compounds	Time (h)	Total motility (mean ± SE of percentage)					
		Concentrations in *μ*g/mL				VC (0.05% DMSO)	PTF (1 mM)
		0	0.5	1	5		
	1	74.8 ± 7.0	76.3 ± 7.8	76.2 ± 8.6	74.7 ± 8.6	74.8 ± 7.0	82.5 ± 6.6
**5a**	4	77.4 ± 3.6	79.8 ± 2.8	79.8 ± 2.6	78.6 ± 4.6	78.2 ± 3.3	85.4 ± 3.8
	24	28.3 ± 6.5	22.7 ± 5.6	25.2 ± 8.0	28.7 ± 7.1	28.0 ± 6.4	30.5 ± 6.6

	1	81.3 ± 1.9	82.0 ± 3.0	85.5 ± 3.3	82.7 ± 2.9	81.3 ± 1.9	88.2 ± 2.0
**5b**	4	77.4 ± 3.6	81.8 ± 1.6	84.2 ± 3.5	80.4 ± 3.3	78.2 ± 3.3	85.4 ± 3.8
	24	28.5 ± 6.4	26.0 ± 5.1	24.2 ± 7.1	24.3 ± 8.2	28.2 ± 6.3	29.7 ± 7.1

	1	81.3 ± 1.9	80.7 ± 1.5	81.7 ± 4.1	85.5 ± 3.1	81.3 ± 1.9	88.2 ± 2.0
**5c**	4	77.4 ± 3.6	80.6 ± 2.2	83.8 ± 0.9	85.4 ± 2.2	78.2 ± 3.3	85.4 ± 3.8
	24	28.3 ± 6.5	23.5 ± 6.7	25.0 ± 7.1	25.0 ± 7.4	28.0 ± 6.4	30.5 ± 6.6

	1	81.3 ± 1.9	80.5 ± 2.0	86.5 ± 2.1	83.3 ± 1.8	81.3 ± 1.9	88.2 ± 2.0
**5d**	4	77.4 ± 3.6	87.0 ± 5.0	86.4 ± 2.6	78.6 ± 3.2	78.2 ± 3.3	85.4 ± 3.8
	24	28.5 ± 6.4	24.2 ± 6.7	25.5 ± 7.5	24.7 ± 9.2	28.2 ± 6.3	29.7 ± 7.1

	1	74.3 ± 6.9	75.5 ± 4.7	75.7 ± 8.8	74.0 ± 9.2	74.5 ± 7.0	81.0 ± 6.2
**5e**	4	70.5 ± 7.5	76.7 ± 5.3	74.3 ± 6.5	74.5 ± 7.8	71.8 ± 7.5	78.7 ± 7.4
	24	28.3 ± 6.5	21.7 ± 6.5	26.7 ± 8.0	27.0 ± 7.6	28.0 ± 6.4	30.5 ± 6.6

	1	76.2 ± 7.5	76.3 ± 7.0	75.8 ± 5.9	72.3 ± 6.7	70.8 ± 7.1	80.0 ± 5.9
**5f**	4	71.7 ± 7.5	77.8 ± 4.9	77.0 ± 7.3	75.3 ± 7.1	70.8 ± 7.8	77.2 ± 7.3
	24	26.8 ± 6.9	25.3 ± 7.3	20.0 ± 6.2	20.2 ± 6.5	18.8 ± 7.1	21.5 ± 7.2

	1	80.3 ± 8.6	81.0 ± 7.8	82.0 ± 6.8	81.0 ± 6.0	80.0 ± 5.9	88.2 ± 3.3
**5g**	4	72.5 ± 7.0	76.7 ± 9.1	72.8 ± 6.5	72.0 ± 8.8	70.2 ± 9.5	80.3 ± 9.1
	24	28.8 ± 4.0	25.6 ± 6.9	25.8 ± 8.0	19.6 ± 6.7	33.6 ± 7.8	31.6 ± 8.5

	1	71.0 ± 11	78.0 ± 6.4	74.0 ± 7.7	77.0 ± 7.1	79.4 ± 4.1	85.0 ± 3.1
**5h**	4	71.8 ± 9.5	75.4 ± 11	69.2 ± 10	76.0 ± 9.9	68.2 ± 12.6	77.4 ± 11
	24	50.8 ± 15	51.3 ± 19	49.8 ± 14	51.0 ± 17	37.0 ± 21.4	45.5 ± 21

	1	74.3 ± 8.5	76.5 ± 6.1	77.7 ± 6.4	77.2 ± 6.3	74.0 ± 6.4	85.2 ± 3.2
**5i**	4	68.7 ± 7.6	69.0 ± 6.7	70.0 ± 8.3	70.5 ± 8.2	66.3 ± 10.2	74.7 ± 8.4
	24	29.4 ± 4.7	43.0 ± 12	28.0 ± 9.9	46.8 ± 12	20.4 ± 8.9	33.2 ± 13

	1	75.3 ± 8.8	78.3 ± 4.8	79.5 ± 5.3	82.3 ± 2.3	78.3 ± 6.0	84.5 ± 3.1
**5j**	4	68.2 ± 7.5	66.5 ± 8.1	68.0 ± 8.3	69.8 ± 8.4	63.0 ± 9.3	71.5 ± 7.3
	24	15.2 ± 6.7	17.4 ± 6.2	20.4 ± 7.8	20.0 ± 5.9	15.2 ± 9.0	19.2 ± 9.2

**(b):** 

Compounds	Time (h)	Progressive motility (mean ± SE of percentage)					
		Concentrations in *μ*g/mL				VC (0.05% DMSO)	PTF (1 mM)
		0	0.5	1	5		
	1	58.8 ± 6.0	58.2 ± 6.9	57.5 ± 7.3	58.3 ± 7.6	57.3 ± 5.5	66.7 ± 6.8
**5a**	4	55.6 ± 4.1	53.2 ± 5.2	56.4 ± 4.9	56.4 ± 5.0	57.6 ± 4.0	69.2 ± 4.2
	24	15.8 ± 4.4	14.5 ± 3.6	16.0 ± 5.4	16.0 ± 4.4	15.2 ± 4.1	18.5 ± 4.7

	1	63.0 ± 3.4	67.8 ± 3.4	67.8 ± 2.9	63.5 ± 2.8	61.5 ± 2.9	73.7 ± 1.8
**5b**	4	55.6 ± 4.1	57.8 ± 3.2	58.8 ± 4.1	58.4 ± 1.8	57.6 ± 4.0	69.2 ± 4.2
	24	16.0 ± 4.3	16.7 ± 3.2	14.8 ± 4.9	12.2 ± 4.4	15.3 ± 4.0	18.0 ± 4.9

	1	63.0 ± 3.4	63.5 ± 1.8	62.0 ± 4.2	69.2 ± 3.4	61.5 ± 2.9	73.7 ± 1.8
**5c**	4	55.6 ± 4.1	60.8 ± 3.9	57.8 ± 2.8	59.0 ± 3.7	57.6 ± 4.0	69.2 ± 4.2
	24	15.8 ± 4.4	16.2 ± 4.6	15.7 ± 4.8	12.8 ± 4.6	15.2 ± 4.1	18.5 ± 4.7

	1	63.0 ± 3.4	64.3 ± 2.4	69.5 ± 2.7	65.2 ± 3.6	61.5 ± 2.9	73.7 ± 1.8
**5d**	4	55.6 ± 4.1	63.2 ± 4.6	63.4 ± 4.3	56.6 ± 7.8	57.6 ± 4.0	69.2 ± 4.2
	24	16.0 ± 4.3	14.3 ± 4.1	14.7 ± 4.1	12.7 ± 5.0	15.3 ± 4.0	18.0 ± 4.9

	1	56.8 ± 5.5	55.5 ± 6.4	58.2 ± 8.0	58.2 ± 6.6	57.0 ± 5.4	66.2 ± 6.6
**5e**	4	60.7 ± 6.0	57.0 ± 5.8	54.5 ± 5.0	53.0 ± 7.3	52.3 ± 6.2	62.7 ± 7.4
	24	15.8 ± 4.4	13.5 ± 4.5	15.2 ± 4.4	15.0 ± 4.6	15.2 ± 4.1	18.5 ± 4.7

	1	53.7 ± 9.2	58.8 ± 8.1	57.3 ± 7.6	54.7 ± 6.6	52.7 ± 9.1	66.8 ± 6.7
**5f**	4	47.5 ± 6.6	53.0 ± 4.0	52.5 ± 6.2	50.0 ± 5.8	46.5 ± 7.2	60.7 ± 6.6
	24	16.5 ± 5.1	18.8 ± 5.4	13.5 ± 5.3	14.8 ± 5.8	10.5 ± 4.2	12.7 ± 4.4

	1	49.0 ± 9.7	59.7 ± 7.0	58.8 ± 7.3	51.5 ± 9.0	54.5 ± 7.8	68.5 ± 2.4
**5g**	4	39.7 ± 8.0	46.0 ± 8.7	42.8 ± 8.0	43.0 ± 10	38.8 ± 8.9	57.3 ± 12
	24	16.0 ± 5.7	14.6 ± 6.9	15.2 ± 5.2	11.8 ± 5.6	17.6 ± 4.7	19.6 ± 5.2

	1	34.2 ± 5.2	45.0 ± 9.3	38.8 ± 10	49.0 ± 6.6	41.8 ± 9.6	58.4 ± 4.1
**5h**	4	53.6 ± 10	58.2 ± 11	48.6 ± 8.8	47.6 ± 8.9	48.4 ± 10.8	56.2 ± 9
	24	29.8 ± 14	31.0 ± 11	23.5 ± 7.5	26.3 ± 10	23.3 ± 15.2	25.0 ± 15

	1	39.2 ± 6.3	45.3 ± 4.2	46.8 ± 6.2	45.7 ± 2.4	42.8 ± 6.2	67.0 ± 4.1
**5i**	4	46.0 ± 6.3	46.2 ± 5.2	46.3 ± 5.8	44.8 ± 6.4	43.3 ± 7.1	58.3 ± 7.2
	24	21.8 ± 4.7	29.2 ± 8.7	20.0 ± 8.4	35.8 ± 10	17.0 ± 8.0	25.6 ± 11

	1	49.0 ± 7.8	47.2 ± 4.1	50.5 ± 5.9	46.8 ± 4.3	52.3 ± 5.9	68.5 ± 4.3
**5j**	4	41.0 ± 5.5	41.5 ± 5.0	40.5 ± 4.7	42.0 ± 5.5	37.0 ± 6.2	58.3 ± 7.2
	24	9.6 ± 4.9	11.2 ± 5.8	12.0 ± 7.1	10.2 ± 5.5	12.4 ± 8.5	13.4 ± 7.9

**Table 3:** Effect of various coumarin derivatives on kinematics of frozen-thawed spermatozoa at one hour after incubation *in vitro*.

Parameters	Compounds	Concentrations of compounds in *µ*g/mL				VC	PTF
		C	0	0.5	5	(0.05% DMSO)	(1 mM)
Velocity curvilinear (VCL)	**5d**	66.2 ± 6.6	64.0 ± 5.3	63.4 ± 6.3	71.0 ± 3.6	66.0 ± 6.6	82.4 ± 5.2
	**5f**	79.5 ± 7.1	80.5 ± 7.4	83.8 ± 7.2	75.5 ± 8.2	77.0 ± 8.1	84.3 ± 7.5
	**5g**	79.8 ± 11.5	75.3 ± 11.2	74.3 ± 6.3	71.0 ± 9.2	78.5 ± 10.6	85.5 ± 5.2
	**5h**	88.5 ± 10.4	79.8 ± 9.0	91.0 ± 2.9	92.0 ± 6.1	85.3 ± 7.3	76.5 ± 10.1
	**5k**	71.8 ± 0.9	71.0 ± 4.7	73.3 ± 4.3	82.5 ± 11.9	70.0 ± 2.9	78.3 ± 5.3

Velocity straight line (VSL)	**5d**	22.2 ± 1.8	22.4 ± 1.0	24.2 ± 1.6	26.6 ± 2.4	22.0 ± 1.8	25.8 ± 2.1
	**5f**	23.8 ± 1.2	23.5 ± 1.0	25.0 ± 1.7	24.5 ± 1.3	23.0 ± 1.7	24.8 ± 2.9
	**5g**	23.0 ± 2.0	24.8 ± 3.5	23.5 ± 4.7	22.8 ± 2.3	26.3 ± 4.6	23.8 ± 2.0
	**5h**	32.8 ± 6.5	31.0 ± 8.1	30.3 ± 4.4	27.3 ± 4.6	30.3 ± 5.6	31.8 ± 6.6
	**5k**	25.5 ± 4.6	24.0 ± 2.6	23.5 ± 3.0	27.5 ± 2.6	25.8 ± 5.0	27.5 ± 3.5

Velocity average Path (VAP)	**5d**	33.4 ± 2.0	33.6 ± 1.7	32.4 ± 1.8	37.6 ± 1.9	33.4 ± 2.0	39.2 ± 1.9
	**5f**	38.5 ± 2.3	40.8 ± 1.9	38.3 **±** 1.4	36.8 ± 2.3	37.8 ± 2.6	40.0 ± 3.0
	**5g**	38.8 ± 4.5	37.0 ± 4.0	39.8 ± 5.0	36.0 ± 4.2	39.5 ± 5.0	40.3 ± 2.8
	**5h**	46.5 ± 7.3	45.5 ± 3.0	43.8 ± 8.0	44.5 ± 3.8	42.8 ± 5.4	44.3 ± 6.2
	**5k**	38.5 ± 2.5	38.5 ± 1.9	37.5 ± 2.4	41.5 ± 4.0	39.0 ± 2.8	39.8 ± 2.9

Amplitude of lateral head displacement (ALH)	**5d**	2.2 ± 0.2	2.2 ± 0.2	2.2 ± 0.2	2.4 ± 0.2	2.2 ± 0.2	3.0 ± 0.3
	**5f**	2.8 ± 0.3	2.8 ± 0.3	2.3 ± 0.5	2.8 ± 0.5	2.3 ± 0.5	2.8 ± 0.5
	**5g**	2.3 ± 0.3	2.3 ± 0.6	2.3 ± 0.5	2.3 ± 0.5	2.5 ± 0.3	3.0 ± 00
	**5h**	2.8 ± 0.3	2.8 ± 0.3	2.8 ± 0.5	3.0 ± 0.4	2.8 ± 0.3	2.0 ± 0.4
	**5k**	2.5 ± 0.3	2.0 ± 0	2.0 ± 00	2.8 ± 0.5	2.0 ± 0.4	2.5 ± 0.5

Linearity (LIN)	**5d**	34.0 ± 1.3	35.6 ± 1.3	38.6 ± 2.9	37.4 ± 2.8	33.8 ± 1.2	31.2 ± 2.3
	**5f**	30.0 ± 1.9	30.5 ± 4.2	30.8 ± 4.5	33.8 ± 4.2	30.0 ± 1.9	29.3 ± 3.1
	**5g**	30.0 ± 2.8	34.0 ± 4.5	30.8 ± 3.8	32.5 ± 1.9	33.3 ± 2.8	42.0 ± 8.1
	**5h**	35.8 ± 3.0	38.3 ± 6.4	33.5 ± 4.6	29.8 ± 4.3	34.3 ± 3.9	42.0 ± 8.1
	**5k**	35.5 ± 6.7	34.0 ± 4.7	33.0 ± 5.2	34.8 ± 4.2	36.8 ± 7.9	35.0 ± 4.1

Straightness (STR)	**5d**	66.0 ± 1.2	69.0 ± 1.7	71.2 ±2.5	66.0 ± 1.2	69.0 ± 1.7	71.2 ±2.5
	**5f**	61.3 ± 3.1	61.3 ± 4.6	61.8 ± 5.1	61.3 ± 3.1	61.3 ± 4.6	61.8 ± 5.1
	**5g**	60.5 ± 3.8	62.8 ± 6.5	61.5 ± 5.1	60.5 ± 3.8	62.8 ± 6.5	61.5 ± 5.1
	**5h**	68.8 ± 3.9	69.3 ± 5.8	65.0 ± 5.1	68.8 ± 3.9	69.3 ± 5.8	65.0 ± 5.1
	**5k**	65.5 ± 7.9	63.8 ± 6.2	61.3 ± 7.5	65.5 ± 7.9	63.8 ± 6.2	61.3 ± 7.5

Wobble (WOB)	**5d**	50.8 ± 2.0	53.6 ± 2.6	51.2 ± 1.6	50.8 ± 2.0	53.6 ± 2.6	51.2 ± 1.6
	**5f**	48.5 ± 1.7	48.8 ± 2.8	49.3 ± 3.1	48.5 ± 1.7	48.8 ± 2.8	49.3 ± 3.1
	**5g**	48.8 ± 1.5	49.5 ± 1.8	53.8 ± 2.2	48.8 ± 1.5	49.5 ± 1.8	53.8 ± 2.2
	**5h**	51.5 ± 2.2	50.8 ± 2.8	54.2 ± 4.8	51.5 ± 2.2	50.8 ± 2.8	54.2 ± 4.8
	**5k**	53.8 ± 4.1	53.0 ± 2.4	53.0 ± 2.4	53.8 ± 4.1	53.0 ± 2.4	53.0 ± 2.4

Beat cross frequency (BCF)	**5d**	8.6 ± 0.5	8.4 ± 0.4	8.4 ± 0.2	8.6 ± 0.5	8.4 ± 0.4	8.4 ± 0.2
	**5f**	9.3 ± 0.6	8.5 ± 1.2	8.8 ± 0.3	9.3 ± 0.6	8.5 ± 1.2	8.8 ± 0.3
	**5g**	10.8 ± 0.9	8.5 ± 1.3	9.3 ± 0.5	10.8 ± 0.9	8.5 ± 1.3	9.3 ± 0.5
	**5h**	10.0 ± 0.5	10.8 ± 0.5	9.3 ± 0.3	10.0 ± 0.5	10.8 ± 0.5	9.3 ± 0.3
	**5k**	9.3 ± 0.3	9.5 ± 0.7	9.8 ± 0.5	9.0 ± 0.4	9.3 ± 0.3	8.8 ± 0.6
