# Supplementary material for: Design and Microwave Assisted Synthesis of Coumarin Derivatives as PDE Inhibitors
Source: Int J Med Chem. 2016 Feb 21;2016:9890630. doi: 10.1155/2016/9890630 (PMC4779523; doi:10.1155/2016/9890630)
Supplement: Supplementary file 1 — The supplementary material available online contains spectral characterization of title compounds 5a-x, copies of IR, 1H-NMR, 13C-NMR, and Mass spectra of compounds 5a, 5d and 5e. [file 9890630.f1.doc]

**Supplementary Material**

Mahadev N. Kumbar^1^, Ravindra R. Kamble^1*^, Atulkumar A. Kamble^1^, Sujith Raj Salian^2^, Sandhya Kumari^2^, Ramya Nair^2^, Guruprasad Kalthur^2*^, Satish Kumar Adiga^2^, D. Jagadeesh Prasad^3^

**Design and Microwave assisted synthesis of coumarin derivatives as PDE inhibitors**

***Corresponding author:** ^1^Department of Chemistry, Karnatak University, Pavate Nagar, Dharwad -580003, Karnataka, India

**E-mail:** [ravichem@kud.ac.in](mailto:ravichem@kud.ac.in)

**Table of Contents**

Spectroscopic data

1. IR Spectrum (KBr) of compound **5a** S1

2. ^1^H NMR spectrum (DMSO-d_6_) of compound **5a** S2

3. ^1^H NMR Expansion Spectrum (DMSO-d6) of compound **5a** S3

4. ^13^C NMR spectrum (DMSO-d6) of compound **5a** S4

5. ^13^C NMR Expansion Spectrum (DMSO-d_6_) of compound **5a** S5

6. Mass Spectrum of compound **5a** S6

7. IR Spectrum (KBr) of compound **5d** S7

8. ^1^H NMR spectrum (DMSO-d_6_) of compound **5d** S8

9. ^1^H NMR Expansion Spectrum (DMSO-d6) of compound **5d** S9

10. ^13^C NMR spectrum (DMSO-d6) of compound **5d** S10

11. ^13^C NMR Expansion Spectrum (DMSO-d_6_) of compound **5d** S11

12. Mass Spectrum of compound **5d**  S12

13. IR Spectrum (KBr) of compound **5e** S13

14. ^1^H NMR spectrum (DMSO-d_6_) of compound **5e** S14

15. ^1^H NMR Expansion Spectrum (DMSO-d6) of compound **5e** S15

16. ^13^C NMR spectrum (DMSO-d6) of compound **5e** S16

17. ^13^C NMR Expansion Spectrum (DMSO-d_6_) of compound **5e** S17

18. Mass Spectrum of compound **5e** S18

*5.1.1 3-(4-(1H-Benzo[d]imidazol-2-yl)-1-phenyl-1H-pyrazol-3-yl)-2H-chromen-2-one* **(5a)**

m.p. 224–226 °C (dec.), IR (KBr, *ν*_max_, cm^-1^): 1711 (coumarin, C=O), 1605 (CN), 3362 (benzimidazole, N-H). ^1^H NMR (400 MHz, DMSO-d_6_): *δ* 7.09 (d, 1H, Ar-H), 7.10 (t, 1H, Ar-H), 7.12 (t, 1H, Ar-H), 7.13 (t, 1H, Ar-H), 7.15 (t, 1H, Ar-H), 7.17 (d, 1H, Ar-H), 7.41-7.49 (m, 5H, Ar-H), 7.59 (d, 1H, Ar-H), 7.69 (d, 1H, Ar-H), 7.74 (s, 1H, coumarin C_4_-H), 8.12 (s, 1H, pyrazole C_3_-H), 12.42 (s, 1H, benzimidazole N-H) ppm. ^13^C NMR (100 MHz, DMSO-d_6_): *δ* 160.76 (coumarin C=O), 158.83 (pyrazole C_3_), 153.60 (benzimidazole C_2_), 145.73, 143.64, 142.70, 138.93, 134.37, 132.25, 129.82, 129.71, 128.88, 128.84, 127.21, 124.73, 122.00, 121.36, 121.24, 119.16, 118.73, 118.73, 118.45, 116.21, 115.03, 110.97 ppm. MS-EI, (*m/z)*: = 404 [M+1]; Anal. for C_25_H_16_N_4_O_2_ (404.4): calcd. C 74.25, H 3.99, N 13.85; Found: C 74.34, H 4.05, N 13.96.

*5.1.2 3-(4-(6-Chloro-1H-benzo[d]imidazol-2-yl)-1-phenyl-1H-pyrazol-3-yl)-2H-chromen-2-one* **(5b)**

m.p. 206–208 °C (dec.), IR (KBr, *ν*_max_, cm^-1^): 1724 (coumarin, C=O), 1619 (CN), 3368 (benzimidazole, N-H). ^1^H NMR (400 MHz, DMSO-d_6_): *δ* 7.05 (d, 1H, Ar-H), 7.09 (d, 1H, Ar-H), 7.11 (t, 1H, Ar-H), 7.13 (t, 1H, Ar-H), 7.15 (d, 1H, Ar-H), 7.39-7.51 (m, 5H, Ar-H), 7.62 (d, 1H, Ar-H), 7.73 (s, 1H, Ar-H), 7.77 (s, 1H, coumarin C_4_-H), 8.69 (s, 1H, pyrazole C_3_-H), 13.09 (s, 1H, benzimidazole N-H) ppm. ^13^C NMR (100 MHz, DMSO-d_6_): *δ* 162.43 (coumarin C=O), 156.52 (pyrazole C_3_), 151.04 (benzimidazole C_2_), 147.53, 145.87, 141.56, 139.31, 135.37, 133.66, 131.09, 130.94, 129.23, 128.64, 126.53, 123.43, 122.72, 120.96, 120.03, 119.16, 118.57, 118.06, 117.32, 116.72, 113.50, 108.54 ppm. MS-EI, (*m/z)*: = 440 [M+2], 438 [M+1]; Anal. for C_25_H_15_N_4_ClO_2_ (438.9): calcd. C 68.42, H 3.45, N 12.77; Found: C 68.51, H 3.53, N 12.87.

*5.1.3 3-(4-(6-Methyl-1H-benzo[d]imidazol-2-yl)-1-phenyl-1H-pyrazol-3-yl)-2H-chromen-2-one* **(5c)**

m.p. 194–196 °C (dec.), IR (KBr, *ν*_max_, cm^-1^): 1716 (coumarin, C=O), 1633 (CN), 3363 (benzimidazole, N-H). ^1^H NMR (400 MHz, DMSO-d_6_): *δ* 2.41 (s, 3H, CH_3_), 7.01 (d, 1H, Ar-H), 7.04 (d, 1H, Ar-H), 7.07 (d, 1H, Ar-H), 7.10 (t, 1H, Ar-H), 7.11 (t, 1H, Ar-H), 7.13 (d, 1H, Ar-H), 7.32-7.56 (m, 5H, Ar-H), 7.64 (s, 1H, Ar-H), 7.78 (s, 1H, coumarin C_4_-H), 8.54 (s, 1H, pyrazole C_3_-H), 12.72 (s, 1H, benzimidazole N-H) ppm. ^13^C NMR (100 MHz, DMSO-d_6_): *δ* 160.67 (coumarin C=O), 154.90 (pyrazole C_3_), 150.24 (benzimidazole C_2_), 148.67, 146.69, 142.90, 138.31, 136.47, 134.18, 133.83, 131.37, 130.35, 129.86, 128.03, 126.53, 123.29, 121.37, 120.61, 118.73, 117.47, 116.82, 115.04, 114.26, 112.71, 106.41, 24.92 (CH_3_) ppm. MS-EI, (*m/z)*: = 418.45 [M+1];. Anal. for C_26_H_18_N_4_O_2_ (418.8): calcd. C 74.63, H 4.34, N 13.39; Found: C 74.75, H 4.41, N 13.46.

*5.1.4 3-(4-(6-Nitro-1H-benzo[d]imidazol-2-yl)-1-phenyl-1H-pyrazol-3-yl)-2H-chromen-2-one* **(5d)**

m.p. 172–174 °C (dec.), IR (KBr, *ν*_max_, cm^-1^): 1725 (coumarin, C=O), 1625 (CN), 3366 (benzimidazole, N-H). ^1^H NMR (400 MHz, DMSO-d_6_): *δ* 6.74 (d, 1H, Ar-H), 7.40 (d, 1H, Ar-H), 7.44 (t, 1H, Ar-H), 7.51 (t, 1H, Ar-H), 7.66 (d, 1H, Ar-H), 7.68-7.98 (m, 5H, Ar-H), 8.29 (d, 1H, Ar-H), 8.47 (s, 1H, Ar-H), 8.64 (s, 1H, coumarin C_4_-H), 8.74 (s, 1H, pyrazole C_3_-H), 11.31 (s, 1H, benzimidazole N-H) ppm. ^13^C NMR (100 MHz, DMSO-d_6_): *δ* 159.70 (coumarin C=O), 153.48 (pyrazole C_3_), 152.79 (benzimidazole C_2_), 150.56, 147.08, 142.76, 138.79, 135.99, 134.60, 132.36, 130.36, 129.69, 128.94, 127.79, 127.36, 124.79, 123.91, 121.71, 120.68, 119.29, 119.00, 118.83, 116.11, 112.61, 112.31 ppm. MS-EI, (*m/z)*: = 449 [M+1]; Anal. for C_25_H_15_N_5_O_4_ (449.4): calcd. C 66.81, H 3.36, N 15.58; Found: C 66.93, H 3.44, N 15.67.

*5.1.5 3-(4-(6-Bromo-3H-imidazo[4,5-b]pyridin-2-yl)-1-phenyl-1H-pyrazol-3-yl)-2H-chromen-2-one* **(5e)**

m.p. 201–203 °C (dec.), IR (KBr, *ν*_max_, cm^-1^): 1718 (coumarin, C=O), 1625 (CN), 3330 (benzimidazole, N-H). ^1^H NMR (400 MHz, DMSO-d_6_): *δ* 6.81 (d, 1H, Ar-H), 7.09 (t, 1H, Ar-H), 7.40 (d, 1H, Ar-H), 7.44 (t, 1H, Ar-H), 7.47-7.61 (m, 5H, Ar-H), 7.70 (s, 1H, Ar-H), 7.87 (s, 1H, coumarin C_4_-H), 7.93 (s, 1H, Ar-H), 8.42 (s, 1H, pyrazole C_3_-H), 11.22 (s, 1H, benzimidazole N-H) ppm. ^13^C NMR (100 MHz, DMSO-d_6_): *δ* 160.25 (coumarin C=O), 158.88 (pyrazole C_3_), 153.93 (benzimidazole C_2_), 153.54, 151.54, 149.03, 145.73, 145.00, 143.64, 142.53, 138.90, 134.11, 132.24, 129.85, 128.85, 127.27, 124.27, 122.40, 119.11, 118.71, 116.13, 115.03, 112.79, 110.97 ppm. MS-EI, (*m/z)*: = 487 [M+2], 484 [M+1]; Anal. for C_24_H_14_N_5_BrO_2_ (484.4): calcd. C 59.52, H 2.91, N 14.46; Found: C 59.64, H 2.99, N 14.55.

*5.1.6 3-(4-(5,7-Dimethyl-1H-benzo[d]imidazol-2-yl)-1-phenyl-1H-pyrazol-3-yl)-2H-chromen-2-one* **(5f)**

m.p. 202–204 °C (dec.), IR (KBr, *ν*_max_, cm^-1^): 1721 (coumarin, C=O), 1629 (CN), 3352 (benzimidazole, N-H). ^1^H NMR (400 MHz, DMSO-d_6_): *δ* 2.39 (s, 3H, CH_3_), 2.41 (s, 3H, CH_3_), 6.89 (s, 1H, Ar-H), 6.94 (d, 1H, Ar-H), 7.05 (d, 1H, Ar-H), 7.09 (t, 1H, Ar-H), 7.13 (t, 1H, Ar-H), 7.32 (s, 1H, Ar-H), 7.44-7.63 (m, 5H, Ar-H), 7.75 (s, 1H, coumarin C_4_-H), 8.41 (s, 1H, pyrazole C_3_-H), 12.60 (s, 1H, benzimidazole N-H) ppm. ^13^C NMR (100 MHz, DMSO-d_6_): *δ* 158.02 (coumarin C=O), 157.31 (pyrazole C_3_), 155.28 (benzimidazole C_2_), 150.56, 148.43, 144.70, 143.89, 140.77, 138.64, 136.39, 134.61, 133.57, 132.49, 131.06, 130.46, 127.76, 125.09, 124.36, 122.09, 121.42, 119.12, 117.43, 115.76, 114.02, 113.78, 25.96, 22.67 (CH_3_)_2_ ppm. MS-EI, (*m/z)*: = 432.16 [M+1]; Anal. for C_27_H_20_N_4_O_2_ (432.5): calcd. C 74.98, H, 4.66, N 12.95; Found: C 75.07, H 4.74, N 12.99.

*5.1.7 3-(4-(1H-Benzo[d]imidazol-2-yl)-1-phenyl-1H-pyrazol-3-yl)-6-chloro-2H-chromen-2-one* **(5g)**

m.p. 176–178 °C (dec.), IR (KBr, *ν*_max_, cm^-1^): 1722 (coumarin, C=O), 1634 (CN), 3345 (benzimidazole, N-H). ^1^H NMR (400 MHz, DMSO-d_6_): *δ* 7.03 (d, 1H, Ar-H), 7.12 (d, 1H, Ar-H), 7.26 (t, 1H, Ar-H), 7.29 (t, 1H, Ar-H), 7.38-7.52 (m, 5H, Ar-H), 7.68 (d, 1H, Ar-H), 7.73 (s, 1H, coumarin C_4_-H), 7.74 (d, 1H, Ar-H), 8.06 (s, 1H, Ar-H), 8.98 (s, 1H, pyrazole C_3_-H), 11.75 (s, 1H, benzimidazole N-H) ppm. ^13^C NMR (100 MHz, DMSO-d_6_): *δ* 162.45 (coumarin C=O), 155.72 (pyrazole C_3_), 154.09 (benzimidazole C_2_), 147.53, 145.72, 141.42, 136.71, 135.91, 130.13, 128.96, 128.73, 127.89, 127.32, 126.82, 125.82, 123.90, 122.32, 120.89, 118.24, 117.07, 116.54, 115.76, 114.09, 114.01, 110.64 ppm. MS-EI, (*m/z)*: = 440 [M+2], 438 [M+1]; Anal. For C_25_H_15_N_4_ClO_2_ (438.9): calcd. C 68.42, H 3.45, N 12.77; Found: C 68.39, H 3.37, N 12.69.

*5.1.8 6-Chloro-3-(4-(6-chloro-1H-benzo[d]imidazol-2-yl)-1-phenyl-1H-pyrazol-3-yl)-2H-chromen-2-one* **(5h)**

m.p. 183–185 °C (dec.), IR (KBr, *ν*_max_, cm^-1^): 1717 (coumarin, C=O), 1614 (CN), 3350 (benzimidazole, N-H). ^1^H NMR (400 MHz, DMSO-d_6_): *δ* 7.09 (d, 1H, Ar-H), 7.14 (d, 1H, Ar-H), 7.27 (s, 1H, Ar-H) 7.29 (d, 1H, Ar-H), 7.30 (d, 1H, Ar-H), 7.35-7.61 (m, 5H, Ar-H), 7.66 (s, 1H, Ar-H), , 7.78 (s, 1H, coumarin C_4_-H), 8.71 (s, 1H, pyrazole C_3_-H), 10.82 (s, 1H, benzimidazole N-H) ppm. ^13^C NMR (100 MHz, DMSO-d_6_): *δ* 159.76 (coumarin C=O), 156.33 (pyrazole C_3_), 152.73 (benzimidazole C_2_), 149.71, 147.61, 144.89, 142.76, 140.21, 137.75, 135.46, 133.87, 131.56, 130.54, 129.07, 127.45, 126.73, 124.32, 122.80, 119.27, 118.34, 115.01, 113.24, 112.11, 111.72, 108.81 ppm. MS-EI, (*m/z)*: = 478 [M+4], 474 [M+2], 472 [M+1]; Anal. for C_25_H_14_N_4_Cl_2_O_2_. (473.3): calcd. C 63.44, H 2.98, N 11.84; Found: C 63.57, H 3.01, N 11.96.

*5.1.9 6-Chloro-3-(4-(6-methyl-1H-benzo[d]imidazol-2-yl)-1-phenyl-1H-pyrazol-3-yl)-2H-chromen-2-one* **(5i)**

m.p. 162–164 °C (dec.), IR (KBr, *ν*_max_, cm^-1^): 1721 (coumarin, C=O), 1617 (CN), 3353 (benzimidazole, N-H). ^1^H NMR (400 MHz, DMSO-d_6_): *δ* 2.41 (s, 3H, CH_3_), 6.69 (d, 1H, Ar-H), 6.89 (d, 1H, Ar-H), 7.16 (d, 1H, Ar-H), 7.29 (s, 1H, Ar-H), 7.53 (s, 1H, Ar-H), 7.55 (d, 1H, Ar-H), 7.56-7.69 (m, 5H, Ar-H), 7.76 (s, 1H, coumarin C_4_-H), 8.79 (s, 1H, pyrazole C_3_-H), 10.42 (s, 1H, benzimidazole N-H) ppm. ^13^C NMR (100 MHz, DMSO-d_6_): *δ* 160.58 (coumarin C=O), 159.31 (pyrazole C_3_), 157.13 (benzimidazole C_2_), 153.70, 151.46, 149.82, 145.71, 142.79, 140.01, 138.73, 136.74, 133.06, 131.58, 130.47, 129.48, 125.79, 124.04, 123.91, 121.27, 119.65, 117.57, 115.53, 113.64, 112.46, 110.42 ppm. MS-EI, (*m/z)*: = 454 [M+2], 452 [M+1]; Anal. for C_25_H_17_N_4_ClO_2_. (452.9): calcd. C 68.95, H 3.78, N 12.37; Found: C 69.07, H 3.91, N 12.47.

*5.1.10 6-Chloro-3-(4-(6-nitro-1H-benzo[d]imidazol-2-yl)-1-phenyl-1H-pyrazol-3-yl)-2H-chromen-2-one* **(5j)**

m.p. 169–171 °C (dec.), IR (KBr, *ν*_max_, cm^-1^): 1734 (coumarin, C=O), 1622 (CN), 3361 (benzimidazole, N-H). ^1^H NMR (400 MHz, DMSO-d_6_): *δ* 6.76 (d, 1H, Ar-H), 7.17 (d, 1H, Ar-H), 7.35 (s, 1H, Ar-H), 7.48 (d, 1H, Ar-H), 7.51-7.64 (m, 5H, Ar-H), 7.81 (s, 1H, coumarin C_4_-H), 8.19 (d, 1H, Ar-H), 8.64 (s, 1H, Ar-H), 8.83 (s, 1H, pyrazole C_3_-H), 11.55 (s, 1H, benzimidazole N-H) ppm. ^13^C NMR (100 MHz, DMSO-d_6_): *δ* 160.79 (coumarin C=O), 155.04 (pyrazole C_3_), 153.16 (benzimidazole C_2_), 149.47, 147.89, 144.66, 142.51, 138.46, 136.15, 134.08, 133.42, 131.42, 130.14, 129.70, 128.40, 126.28, 125.06, 123.47, 121.41, 120.27, 119.43, 117.95, 114.72, 113.08, 109.39 ppm. MS-EI, (*m/z)*: = 485 [M+2], 483 [M+1]; Anal. for C_25_H_14_N_5_ClO_4_ (483.9): calcd. C 62.06, H 2.92, N 14.47; Found: C 62.12, H 3.01, N 14.59.

*5.1.11 3-(4-(6-Bromo-1H-imidazo[4,5-b]pyridin-2-yl)-1-phenyl-1H-pyrazol-3-yl)-6-chloro2H-chromen-2-one* **(5k)**

m.p. 186–188 °C (dec.), IR (KBr, *ν*_max_, cm^-1^): 1723 (coumarin, C=O), 1629 (CN), 3354 (benzimidazole, N-H). ^1^H NMR (400 MHz, DMSO-d_6_): *δ* 6.98 (d, 1H, Ar-H), 7.14 (d, 1H, Ar-H), 7.29 (s, 1H, Ar-H), 7.34-7.47 (m, 5H, Ar-H), 7.69 (s, 1H, Ar-H), 7.92 (s, 1H, coumarin C_4_-H), 8.46 (s, 1H, Ar-H), 8.80 (s, 1H, pyrazole C_5_-H), 12.73 (s, 1H, benzimidazole N-H) ppm. ^13^C-NMR (100 MHz, DMSO-d6): *δ* 159.01 (coumarin C=O), 157.76 (pyrazole C_3_), 152.11 (benzimidazole C_2_), 150.37, 149.03, 145.56, 143.47, 141.75, 139.79, 136.75, 133.87, 131.80, 129.34, 126.13, 126.68, 124.06, 122.39, 120.43, 119.72, 116.52, 116.02, 115.59, 112.69, 108.94 ppm. MS-EI, (*m/z)*: = 525 [M+4], 521.0, [M+2], 518 [M+1]; Anal. for C_24_H_13_N_5_BrClO_2_ (518.7): calcd. C 55.57, H 2.53, N 13.50; Found: C 55.69, H 2.68, N 13.63.

*5.1.12 6-Chloro-3-(4-(5,7-dimethyl-1H-benzo[d]imidazol-2-yl)-1-phenyl-1H-pyrazol-3-yl)-2H-chromen-2-one* **(5l)**

m.p. 167–169 °C (dec.), IR (KBr, *ν*_max_, cm^-1^): 1715 (coumarin, C=O), 1629 (CN), 3346 (benzimidazole, N-H). ^1^H NMR (400 MHz, DMSO-d_6_): *δ* 2.39 (s, 3H, CH_3_), 2.47 (s, 3H, CH_3_), 6.91 (s, 1H, Ar-H), 7.08 (d, 1H, Ar-H), 7.16 (d, 1H, Ar-H), 7.29 (s, 1H, Ar-H), 7.33 (s, 1H, Ar-H), 7.39-7.52 (m, 5H, Ar-H), 7.77 (s, 1H, coumarin C_4_-H), 8.26 (s, 1H, pyrazole C_3_-H), 12.82 (s, 1H, benzimidazole N-H) ppm. ^13^C NMR (100 MHz, DMSO-d_6_): *δ* 161.07 (coumarin C=O), 159.35 (pyrazole C_3_), 157.06 (benzimidazole C_2_), 154.52, 150.44, 147.36, 146.80, 144.29), 142.68, 140.35, 138.30, 136.08, 135.19, 133.63, 131.60, 129.72, 127.82, 126.08, 124.50, 122.34, 120.43, 118.24, 117.71, 113.37, 110.58, 24.87, 21.46 (CH_3_, CH_3_) ppm. MS-EI, (*m/z)*: = 468 [M+2], 466 [M+1]; Anal. for C_27_H_19_N_4_ClO_2_ (466.9): calcd. C 69.45, H 4.10, N 12.00; Found: C 69.59, H 4.17, N 12.08.

*5.1.13 3-(4-(1H-Benzo[d]imidazol-2-yl)-1-phenyl-1H-pyrazol-3-yl)-6-bromo-2H-chromen-2-one* **(5m)**

m.p. 171–173 °C (dec.), IR (KBr, *ν*_max_, cm^-1^): 1727 (coumarin, C=O), 1637 (CN), 3352 (benzimidazole, N-H). ^1^H NMR (400 MHz, DMSO-d_6_): *δ* 7.07 (d, 1H, Ar-H), 7.15 (d, 1H, Ar-H), 7.22 (t, 1H, Ar-H), 7.27 (t, 1H, Ar-H), 7.35 (d, 1H, Ar-H), 7.39-7.57 (m, 5H, Ar-H), 7.62 (d, 1H, Ar-H), 7.73 (s, 1H, coumarin C_4_-H), 8.26 (s, 1H, Ar-H), 8.74 (s, 1H, pyrazole C_3_-H), 12.79 (s, 1H, benzimidazole N-H) ppm. ^13^C NMR (100 MHz, DMSO-d_6_): *δ* 161.44 (coumarin C=O), 158.73 (pyrazole C_3_), 155.03 (benzimidazole C_2_), 152.50, 143.76, 139.72, 138.31, 136.58, 134.72, 130.90, 129.03, 128.35, 127.80, 126.00, 124.12, 123.74, 121.38, 120.62, 119.76, 118.27, 117.51, 116.06, 115.27, 112.61, 108.38 ppm. MS-EI, (*m/z)*: = 484 [M+2], 482 [M+1]; Anal. for C_25_H_15_N_4_BrO_2_ (483.3): calcd C 62.13, H 3.13, N 11.59; Found: C 62.22, H 3.20, N 11.65.

*5.1.14 6-Bromo-3-(4-(6-chloro-1H-benzo[d]imidazol-2-yl)-1-phenyl-1H-pyrazol-3-yl)-2H-chromen-2-one* **(5n)**

m.p. 178–180 °C (dec.), IR (KBr, *ν*_max_, cm^-1^): 1732 (coumarin, C=O), 1632 (CN), 3353 (benzimidazole, N-H). ^1^H NMR (400 MHz, DMSO-d_6_): *δ* 7.11 (d, 1H, Ar-H), 7.17 (d, 1H, Ar-H), 7.24 (d, 1H, Ar-H), 7.28 (d, 1H, Ar-H), 7.42-7.59 (m, 5H, Ar-H), 7.64 (s, 1H, Ar-H), 7.76 (s, 1H, coumarin C_4_-H), 8.32 (s, 1H, Ar-H), 8.58 (s, 1H, pyrazole C_3_-H), 11.72 (s, 1H, benzimidazole N-H) ppm. ^13^C NMR (100 MHz, DMSO-d_6_): *δ* 160.71 (coumarin C=O), 157.32 (pyrazole C_3_), 151.04 (benzimidazole C_2_), 149.74, 146.89, 143.41, 142.05, 139.71, 136.49, 134.03, 132.37, 131.05, 130.46, 128.48, 128.00, 125.29, 123.81, 121.42, 119.62, 117.96, 114.16, 112.53, 111.07, 110.12, 108.74 ppm. MS-EI, (*m/z)*: = 524 [M+4], 520, [M+2], 518 [M+1]; Anal. for C_25_H_14_N_4_BrClO_2_(517.8): calcd C 57.99, H 2.73, N 10.82; Found: C 58.08, H 2.81, N 10.94.

*5.1.15 6-Bromo-3-(4-(6-methyl-1H-benzo[d]imidazol-2-yl)-1-phenyl-1H-pyrazol-3-yl)-2H-chromen-2-one* **(5o)**

m.p. 168–170 °C (dec.), IR (KBr, *ν*_max_, cm^-1^): 1737 (coumarin, C=O), 1632 (CN), 3349 (benzimidazole, N-H). ^1^H NMR (400 MHz, DMSO-d_6_): *δ* 2.41 (s, 3H, CH_3_), 6.72 (d, 1H, Ar-H), 7.28 (d, 1H, Ar-H), 7.39 (d, 1H, Ar-H), 7.42 (s, 1H, Ar-H), 7.46 (d, 1H, Ar-H), 7.48-7.63 (m, 5H, Ar-H), 7.68 (s, 1H, Ar-H),7.79 (s, 1H, coumarin C_4_-H), 8.82 (s, 1H, pyrazole C_3_-H), 12.66 (s, 1H, benzimidazole N-H) ppm. ^13^C NMR (100 MHz, DMSO-d_6_): *δ* 161.84 (coumarin C=O), 158*.*47 (pyrazole C_3_), 155.18 (benzimidazoel C_2_), 154.47, 152.72, 146.84, 144.26, 142.01, 140.75, 138.39, 135.38, 133.71, 131.40, 128.03, 127.78, 125.73, 122.14, 121.96, 118.46, 117.85, 116.27, 112.54, 111.06, 108.37, 24.39 -CH_3_ ppm. MS-EI, (*m/z)*: = 498 [M+2], 496 [M+1]; Anal. for C_25_H_17_N_4_BrO_2_(496.3): calcd C 62.79, H 3.45, N 11.27; Found: C 62.86, H 3.56, N 11.36.

*5.1.16 6-Bromo-3-(4-(6-nitro-1H-benzo[d]imidazol-2-yl)-1-phenyl-1H-pyrazol-3-yl)-2H-chromen-2-one* **(5p)**

m.p. 185–187 °C (dec.), IR (KBr, *ν*_max_, cm^-1^): 1730 (coumarin, C=O), 1642 (CN), 3356 (benzimidazole, N-H). ^1^H NMR (400 MHz, DMSO-d_6_): *δ* 6.89 (d, 1H, Ar-H), 7.31 (d, 1H, Ar-H), 7.39 (d, 1H, Ar-H), 7.44 (s, 1H, Ar-H), 7.47-7.68 (m, 5H, Ar-H), 7.81 (s, 1H, coumarin C_4_-H), 8.39 (d, 1H, Ar-H), 8.57 (s, 1H, Ar-H), 8.80 (s, 1H, pyrazole C_3_-H), 12.60 (s, 1H, benzimidazole N-H) ppm. ^13^C NMR (100 MHz, DMSO-d_6_): *δ* 162.86 (coumarin C=O), 158.44 (pyrazole C_3_), 156.05 (benzimidazole C_2_), 153.08, 151.36, 147.63, 145.73, 142.42, 140.55, 138.35, 135.05, 133.22, 131.63, 130.77, 128.14, 125.29, 124.72, 122.16, 120.28, 119.24, 118.78, 116.46, 113.36, 112.43, 110.17 ppm. MS-EI, (*m/z)*: = 535 [M+4], 531 [M+2], 529 [M+1]; Anal. for C_25_H_14_N_5_BrO_4_ (528.3): calcd. C 56.84, H 2.67, N 13.26; Found: C 56.92, H 2.79, N 13.33.

*5.1.17 6-Bromo-3-(4-(6-bromo-1H-imidazo[4,5-b]pyridin-2-yl)-1-phenyl-1H-pyrazol-3-yl)-2H-chromen-2-one* **(5q)**

m.p. 163–165 °C (dec.), IR (KBr, *ν*_max_, cm^-1^): 1720 (coumarin, C=O), 1638 (CN), 3366 (benzimidazole, N-H). ^1^H NMR (400 MHz, DMSO-d_6_): *δ* 7.04 (d, 1H, Ar-H), 7.17 (d, 1H, Ar-H), 7.27 (s, 1H, Ar-H), 7.36-7.52 (m, 5H, Ar-H), 7.72 (s, 1H, coumarin C_4_-H), 7.88 (s, 1H, Ar-H), 8.58 (s, 1H, Ar-H), 8.89 (s, 1H, pyrazole C_3_-H), 13.36 (s, 1H, benzimidazole N-H) ppm. ^13^C NMR (100 MHz, DMSO-d_6_): *δ* 160.23 (coumarin C=O), 155.00 (pyrazole C_3_), 153.64 (benzimidazole C_2_), 151.37, 149.62, 147.18, 144.78, 142.43, 140.26, 138.43, 136.89, 133.31, 130.31, 128.89, 127.62, 125.73, 124.56, 122.75, 120.75, 118.24, 115.17, 113.63, 112.07, 110.44ppm. MS-EI, (*m/z)*: = 569 [M+4], 565 [M+2], 563 [M+1]; Anal. for C_24_H_13_N_5_Br_2_O_2_ (563.2): calcd. C 51.18, H 2.33, N 12.43; Found: C 51.24, H 2.41, N 12.55.

*5.1.18 6-Bromo-3-(4-(5,7-dimethyl-1H-benzo[d]imidazol-2-yl)-1-phenyl-1H-pyrazol-3-yl)-2H-chromen-2-one* **(5r)**

m.p. 174–176 °C (dec.), IR (KBr, *ν*_max_, cm^-1^): 1722 (coumarin, C=O), 1644 (CN), 3357 (benzimidazole, N-H). ^1^H NMR (400 MHz, DMSO-d_6_): *δ* 2.39 (s, 3H, CH_3_), 2.44 (s, 3H, CH_3_), 6.94 (s, 1H, Ar-H), 7.12 (d, 1H, Ar-H), 7.21 (d, 1H, Ar-H), 7.27 (s, 1H, Ar-H), 7.36 (s, 1H, Ar-H), 7.40-7.56 (m, 5H, Ar-H), 7.69 (s, 1H, coumarin C_4_-H), 8.32 (s, 1H, pyrazole C_3_-H), 13.09 (s, 1H, benzimidazole N-H) ppm. ^13^C NMR (100 MHz, DMSO-d_6_): *δ* 158.87 (coumarin C=O), 157.43 (pyrazole C_3_), 156.65 (benzimidazole C_2_), 155.98, 153.67, 149.53, 147.72, 145.88, 141.54, 140.42, 137.29, 136.45, 134.54, 132.60, 130.68, 128.73, 127.22, 125.32, 122.87, 121.04, 118.29, 116.76, 115.35, 112.68, 109.54 , 26.89, 22.57 (CH_3_, CH_3_) ppm. MS-EI, (*m/z)*: = 512 [M+2], 510 [M+1]; Anal. for C_27_H_19_N_4_BrO_2_(511.4): calcd C 63.42, H 3.75, N 10.96; Found: C 63.56, H 3.83, N 11.06.

*5.1.19 3-(4-(1H-Benzo[d]imidazol-2-yl)-1-phenyl-1H-pyrazol-3-yl)-8-methoxy-2H-chromen-2-one* **(5s)**

m.p. 175–177 °C (dec.), IR (KBr, *ν*_max_, cm-1): 1726 (coumarin, C=O), 1640 (CN), 3349 (benzimidazol, N-H). 1H NMR (400 MHz, DMSO-d6): *δ* 3.79 (s, 3H, OCH3), 6.69 (d, 1H, Ar-H), 6.87 (d, 1H, Ar-H), 6.96 (t, 1H, Ar-H), 7.29-7.71 (m, 4H, Ar-H), 7.42-7.78 (m, 5H, Ar-H), 7.84 (s, 1H, coumarin C4-H), 8.74 (s, 1H, pyrazole C3-H), 12.79 (s, 1H, benzimidazole N-H) ppm. 13C NMR (100 MHz, DMSO-d6): *δ* 163.77 (coumarin C=O), 159.74 (pyrazole C3), 156.67 (benzimidazole C2), 154.25, 142.67, 140.34, 139.92, 135.18, 132.35, 131.93, 129.45, 128.42, 127.46, 126.35, 125.87, 122.13, 121.56, 120.80, 119.37, 118.48, 116.91, 115.16, 113.67, 110.26, 109.82), 60.74 (OCH_3_) ppm. MS-EI, (*m/z)*: = 434 [M+1]; Anal. for C_26_H_18_N_4_O_3_ (434.4): calcd. C 71.88, H 4.18, N 12.90; Found: C 71.95, H 4.29, N 12.99.

*5.1.20 3-(4-(6-Chloro-1H-benzo[d]imidazol-2-yl)-1-phenyl-1H-pyrazol-3-yl)-8-methoxy-2H-chromen-2-one* **(5t)**

m.p. 170–172 °C (dec.), IR (KBr, *ν*_max_, cm^-1^): 1732 (coumarin, C=O), 1631 (CN), 3351 (benzimidazole, N-H). ^1^H NMR (400 MHz, DMSO-d_6_): *δ* 3.82 (s, 3H, OCH_3_), 6.67 (d, 1H, Ar-H), 6.84 (d, 1H, Ar-H), 6.94 (t, 1H, Ar-H), 7.29 (d, 1H, Ar-H), 7.40-7.62 (m, 5H, Ar-H), 7.68 (d, 1H, Ar-H), 7.74 (s, 1H, coumarin C_4_-H), 7.76 (s, 1H, pyrazole C_3_-H), 8.14 (s, 1H, Ar-H), 11.36 (s, 1H, benzimidazole N-H) ppm. ^13^C NMR (100 MHz, DMSO-d_6_): *δ* 163.80 (coumarin C=O), 158.15 (pyrazole C_3_), 155.37 (benzimidazole C_2_), 151.26, 144.77, 142.59, 140.24, 138.27, 134.38, 132.03, 130.12, 127.44, 126.85, 125.79, 123.68, 122.31, 121.00, 120.84, 118.35, 116.60, 115.71, 114.42, 112.18, 111.23, 108.44, 61.78 (OCH_3_) ppm. MS-EI, (*m/z)*: = 470 [M+2], 468 [M+1]; Anal. for C_26_H_17_N_4_ClO_3_ (468.9): calcd. C 66.60, H 3.65, N 11.95; Found: C 66.72, H 3.71, N 12.07.

*5.1.21 8-Methoxy-3-(4-(6-methyl-1H-benzo[d]imidazol-2-yl)-1-phenyl-1H-pyrazol-3-yl)-2H-chromen-2-one* **(5u)**

m.p. 182–184 °C (dec.), IR (KBr, *ν*_max_, cm^-1^): 1736 (coumarin, C=O), 1629 (CN), 3357 (benzimidazole, N-H). ^1^H NMR (400 MHz, DMSO-d_6_): *δ* 2.37 (s, 3H, CH_3_), 3.82 (s, 3H, OCH_3_), 6.69 (d, 1H, Ar-H), 6.89 (d, 1H, Ar-H), 6.97 (t, 1H, Ar-H), 7.27 (d, 1H, Ar-H), 7.38-7.56 (m, 5H, Ar-H), 7.66 (d, 1H, Ar-H), 7.70 (s, 1H, coumarin C_4_-H), 7.75 (s, 1H, pyrazole C_3_-H), 8.06 (s, 1H, Ar-H), 12.84 (s, 1H, benzimidazole N-H) ppm. ^13^C NMR (100 MHz, DMSO-d_6_): *δ* 164.74 (coumarin C=O), 156.93 (pyrazole C_3_), 154.69 (benzimidazole C_2_), 152.70, 146.46, 144.89, 143.27, 140.78, 136.32, 134.15, 132.56, 129.47, 128.02, 126.90, 124.37, 123.72, 120.66, 119.87, 118.00, 114.74, 112.91, 112.04, 111.48, 110.53, 109.48, 59.37 (OCH_3_), 25.62 (CH_3_) ppm. MS-EI, (*m/z)*: = 448 [M+1]; Anal. for C_27_H_20_N_4_O_3_ (448.5): calcd. C 72.31, H 4.49, N 12.49; Found: C 72.43, H 4.57, N 12.59.

*5.1.22 8-Methoxy-3-(4-(6-nitro-1H-benzo[d]imidazol-2-yl)-1-phenyl-1H-pyrazol-3-yl)-2H-chromen-2-one* **(5v)**

m.p. 184–186°C (dec.), IR (KBr, *ν*_max_, cm^-1^): 1729 (coumarin, C=O), 1619 (CN), 3348 (benzimidazole, N-H). ^1^H NMR (400 MHz, DMSO-d_6_): *δ* 3.82 (s, 3H, OCH_3_), 6.71 (d, 1H, Ar-H), 6.87 (d, 1H, Ar-H), 6.98 (t, 1H, Ar-H), 7.33 (d, 1H, Ar-H), 7.42-7.66 (m, 5H, Ar-H), 7.71 (d, 1H, Ar-H), 7.75 (s, 1H, coumarin C_4_-H), 7.79 (s, 1H, pyrazole C_3_-H), 8.21 (s, 1H, Ar-H), 12.37 (s, 1H, benzimidazole N-H) ppm. ^13^C NMR (100 MHz, DMSO-d_6_): *δ* 164.77 (coumarin C=O), 156.47 (pyrazole C_3_), 153.28 (benzimidazole C_2_), 150.68, 142.65, 142.03, 141.38, 139.86, 137.47, 136.28, 133.54, 129.98, 127.38, 126.46, 124.71, 122.34, 121.65, 119.43, 117.48, 116.95, 115.39, 113.67, 111.38, 110.48, 108.83, 62.74 (OCH_3_) ppm. MS-EI, (*m/z)*: = 479 [M+1]; Anal. for C_26_H_17_N_5_O_5_. (479.4): calcd. C 65.13, H 3.57, N 14.61; Found: C 65.24, H 3.64, N 14.73.

*5.1.23 3-(4-(6-Bromo-1H-imidazo[4,5-b]pyridin-2-yl)-1-phenyl-1H-pyrazol-3-yl)-8-methoxy-2H chromen-2-one* **(5w)**

m.p. 201–203 °C (dec.), IR (KBr, *ν*_max_, cm^-1^): 1718 (coumarin, C=O), 1619 (CN), 3365 (benzimidazole, N-H). ^1^H NMR (400 MHz, DMSO-d_6_): *δ* 7.03 (d, 1H, Ar-H), 7.07 (t, 1H, Ar-H), 7.13 (t, 1H, Ar-H), 7.44-7.58 (m, 5H, Ar-H), 7.70 (s, 1H, coumarin C_4_-H), 8.16 (s, 1H, Ar-H), 8.27 (s, 1H, Ar-H), 8.54 (s, 1H, pyrazole C_3_-H), 8.82 (s, 1H, Ar-H), 11.89 (s, 1H, benzimidazole N-H) ppm. ^13^C NMR (100 MHz, DMSO-d_6_): *δ* 158.52 (coumarin C=O), 154.27 (pyrazole C_3_), 149.27 (benzimidazole C_2_), 148.31, 146.07, 142.53, 140.79, 137.59, 136.15, 133.04, 132.47, 130.17, 127.39, 125.18, 124.67, 123.41, 121.76, 119.41, 118.02, 117.57, 116.32, 115.50, 114.63, 111.39 ppm. MS-EI, (*m/z)*: = 487 [M+2], 485 [M+1]; Anal. for C_24_H_14_N_5_BrO_2_ (514.3): calcd. C 59.52, H 2.91, N 14.46; Found: C 59.61, H 3.03, N 14.59.

*5.1.24 8-Methoxy-3-(4-(5,7-dimethyl-1H-benzo[d]imidazol-2-yl)-1-phenyl-1H-pyrazol-3-yl)-2H-chromen-2-one* **(5x)**

m.p. 192–194 °C (dec.), IR (KBr, *ν*_max_, cm^-1^): 1719 (coumarin, C=O), 1635 (CN), 3359 (benzimidazole, N-H). ^1^H NMR (400 MHz, DMSO-d_6_): *δ* 2.41 (s, 3H, CH_3_), 2.47 (s, 3H, CH_3_), 3.80 (s, 3H, OCH_3_), 6.65 (d, 1H, Ar-H), 6.84 (d, 1H, Ar-H), 6.68 (s, 1H, Ar-H), 6.93 (t, 1H, Ar-H), 7.34 (s, 1H, Ar-H), 7.36-7.59 (m, 5H, Ar-H), 7.78 (s, 1H, coumarin C_4_-H), 8.06 (s, 1H, pyrazole C_3_-H), 13.08 (s, 1H, benzimidazole N-H) ppm. ^13^C NMR (100 MHz, DMSO-d_6_): *δ* 161.35 (coumarin C=O), 158.07 (pyrazole C_3_), 156.05 (benzimidazole C_2_), 151.24, 145.29, 144.75, 142.63, 141.74, 139.38, 133.74, 130.64, 129.12, 127.18, 127.88, 125.74, 121.52, 120.79, 119.65, 118.37, 116.38, 113.81, 112.64, 110.96, 110.31, 108.63, 58.42 (OCH_3_), 25.84, 21.38 (CH_3_)_2_ ppm. MS-EI, (*m/z)*: = 462 [M+1]; Anal. for C_28_H_22_N_4_O_3_ (462.5): calcd. C 72.71, H 4.79, N 12.11; Found: C 72.83, H 4.86; N 12.19.

**
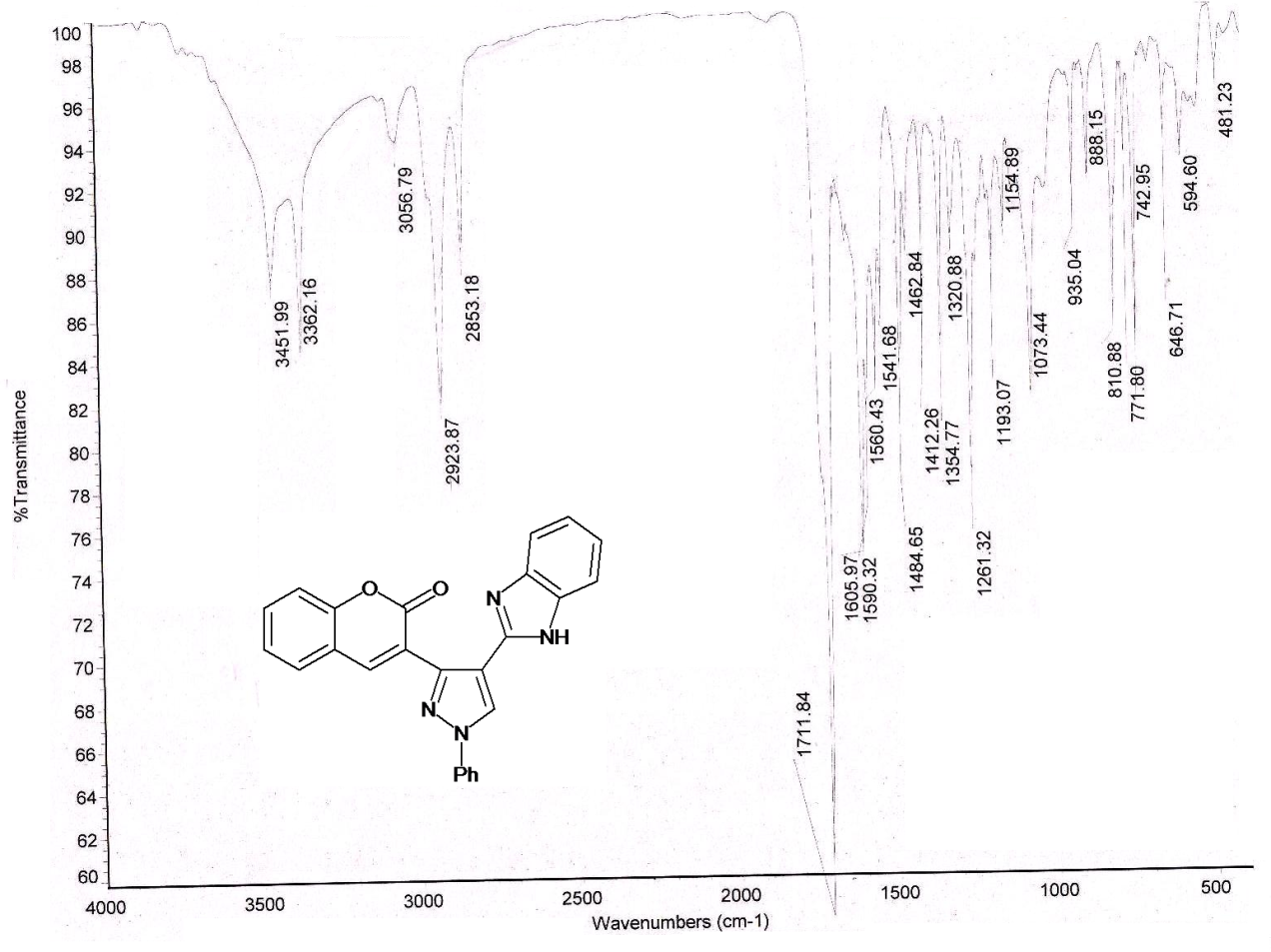
**

**Figure S1. IR Spectrum (KBr) of compound 5a**


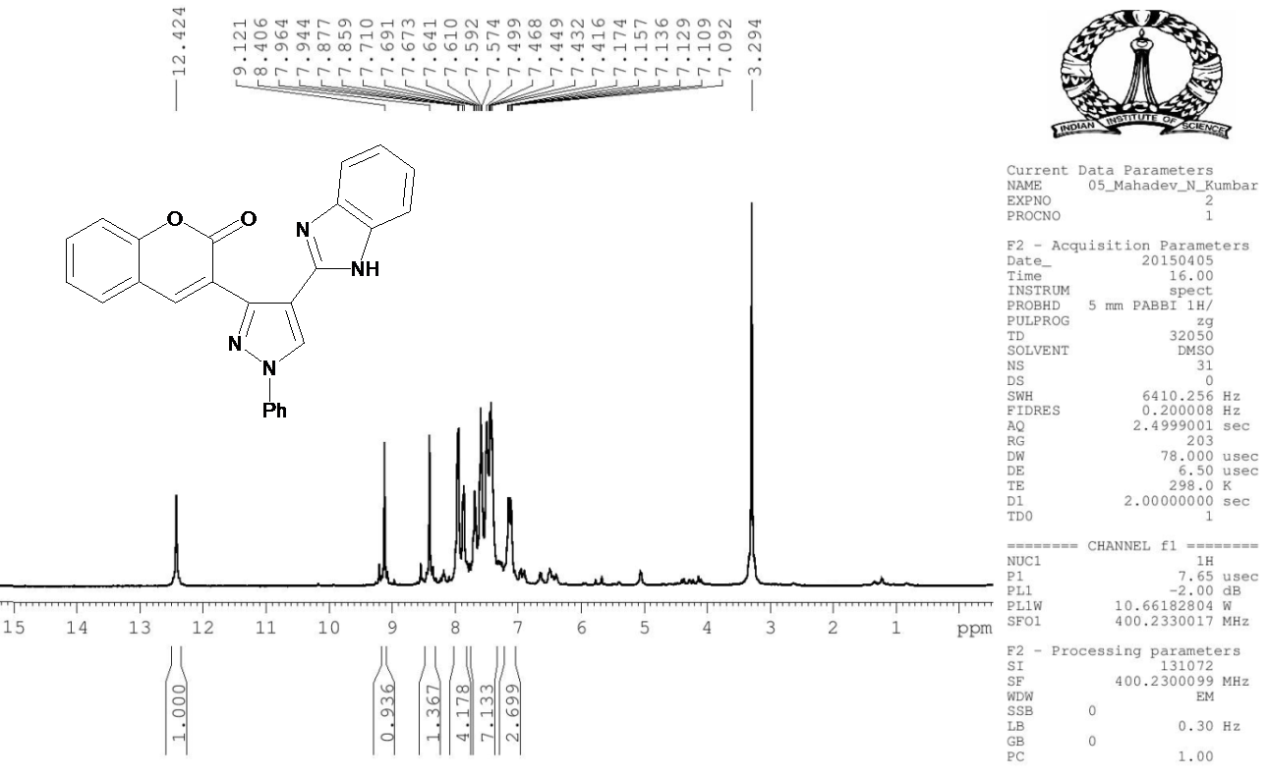


**Figure S2. ^1^H NMR spectrum (DMSO-d_6_) of compound 5a**

**
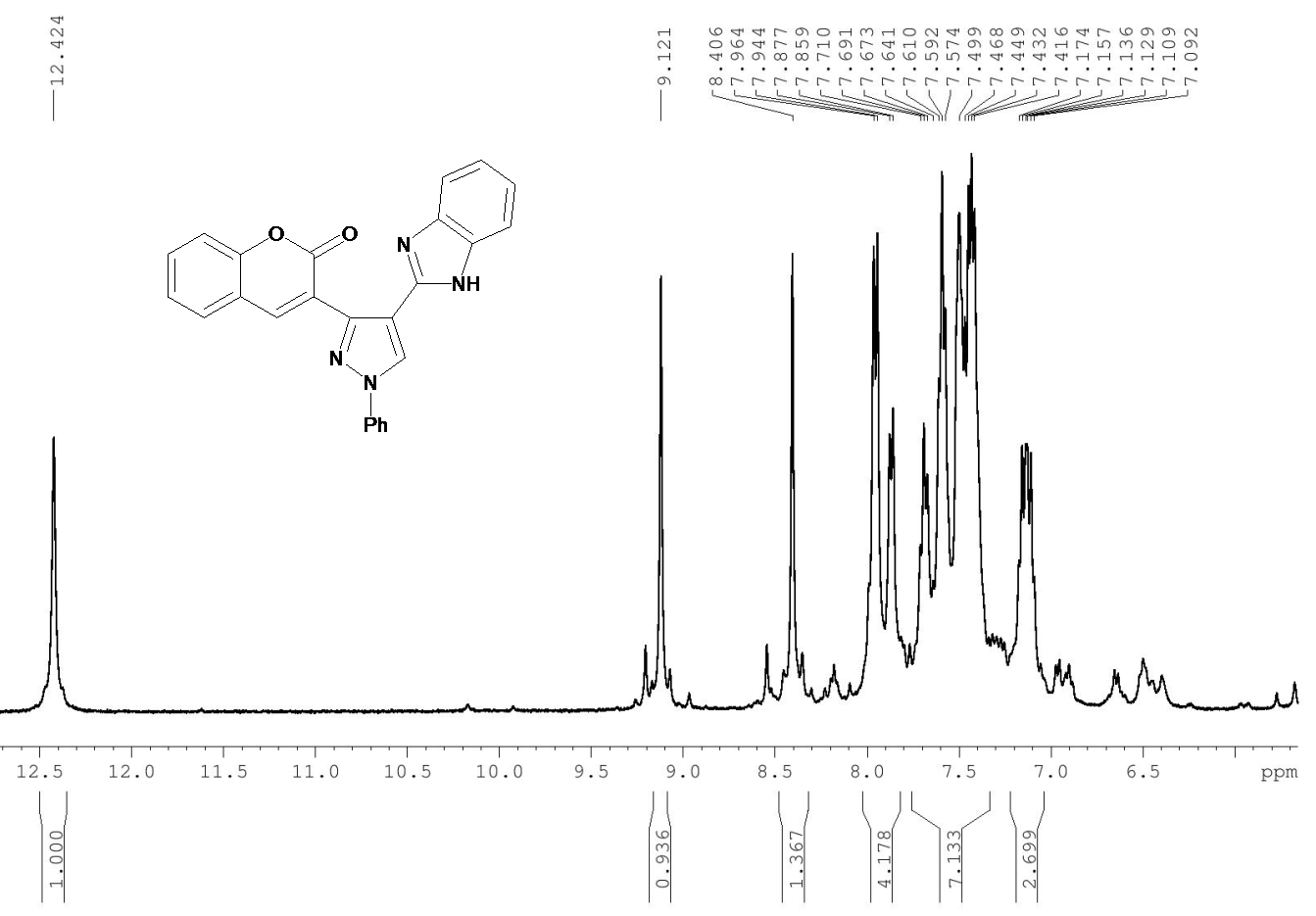
**

**Figure S3. ^1^H NMR Expansion Spectrum (DMSO-d6) of Compound 5a**

**
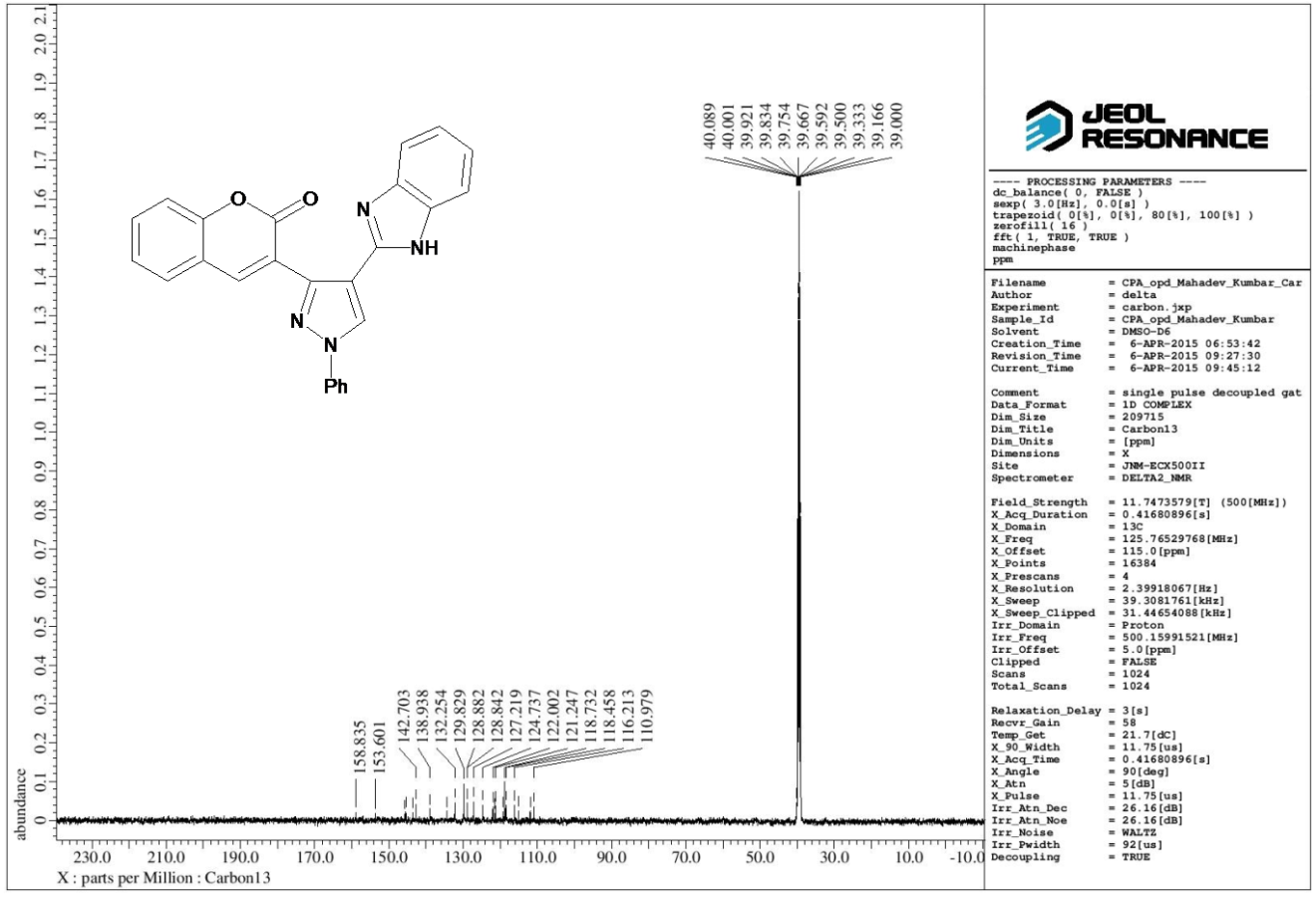
**

**Figure S4. ^13^C NMR spectrum (DMSO-d6) of compound 5a**

**
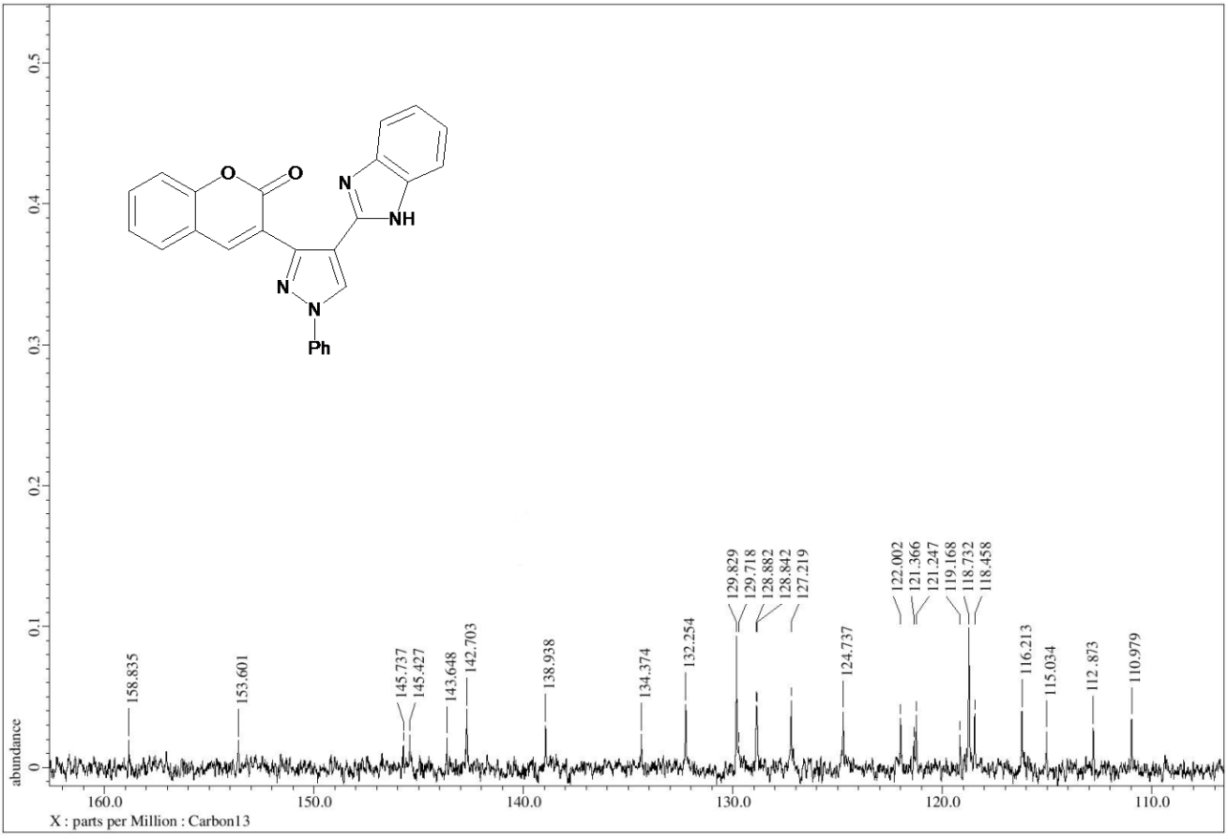
**

**Figure S5. ^13^C NMR Expansion Spectrum (DMSO-d_6_) of Compound 5a**

**
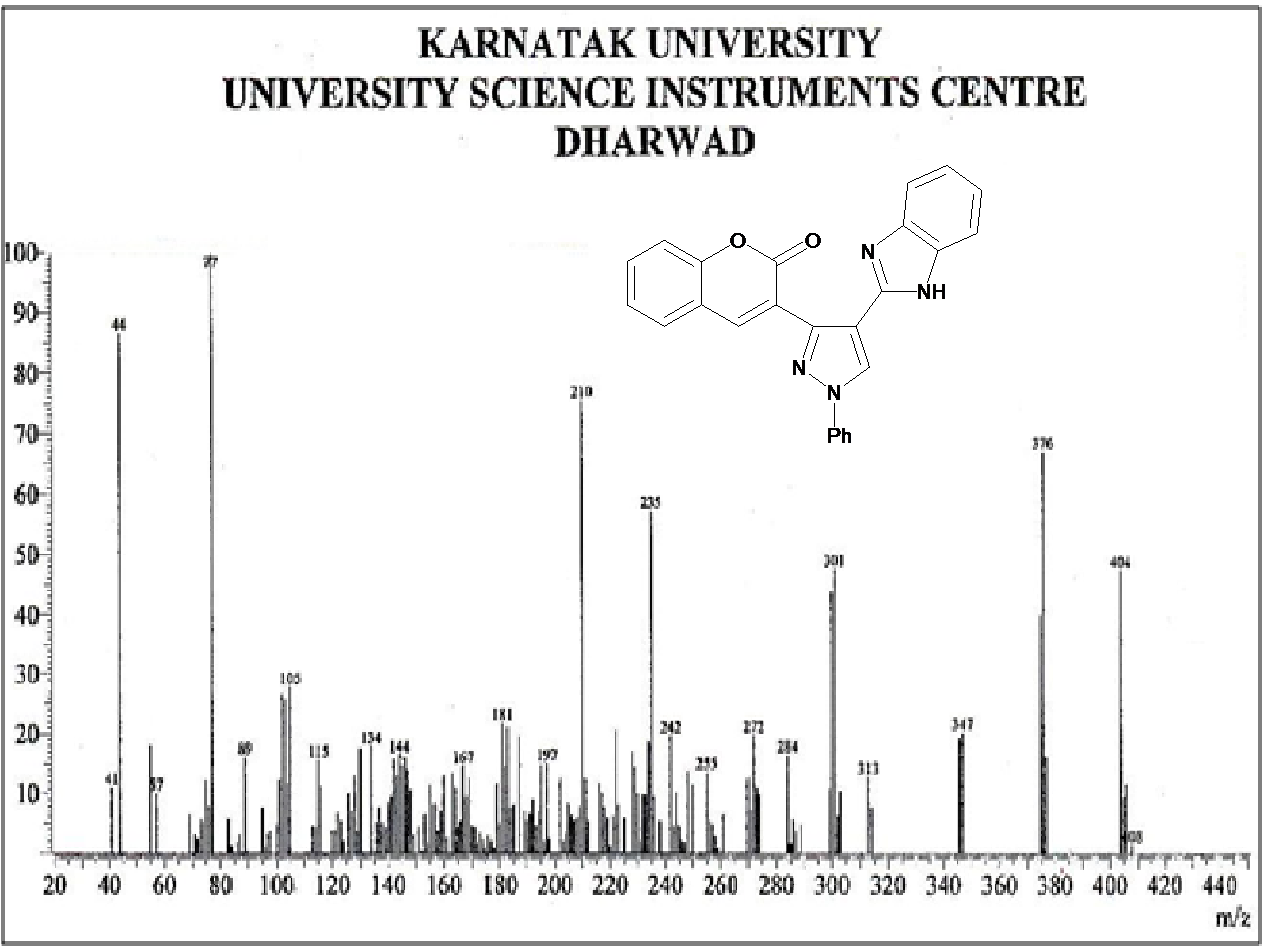
**

**Figure S6. Mass Spectrum of compound 5a**

**
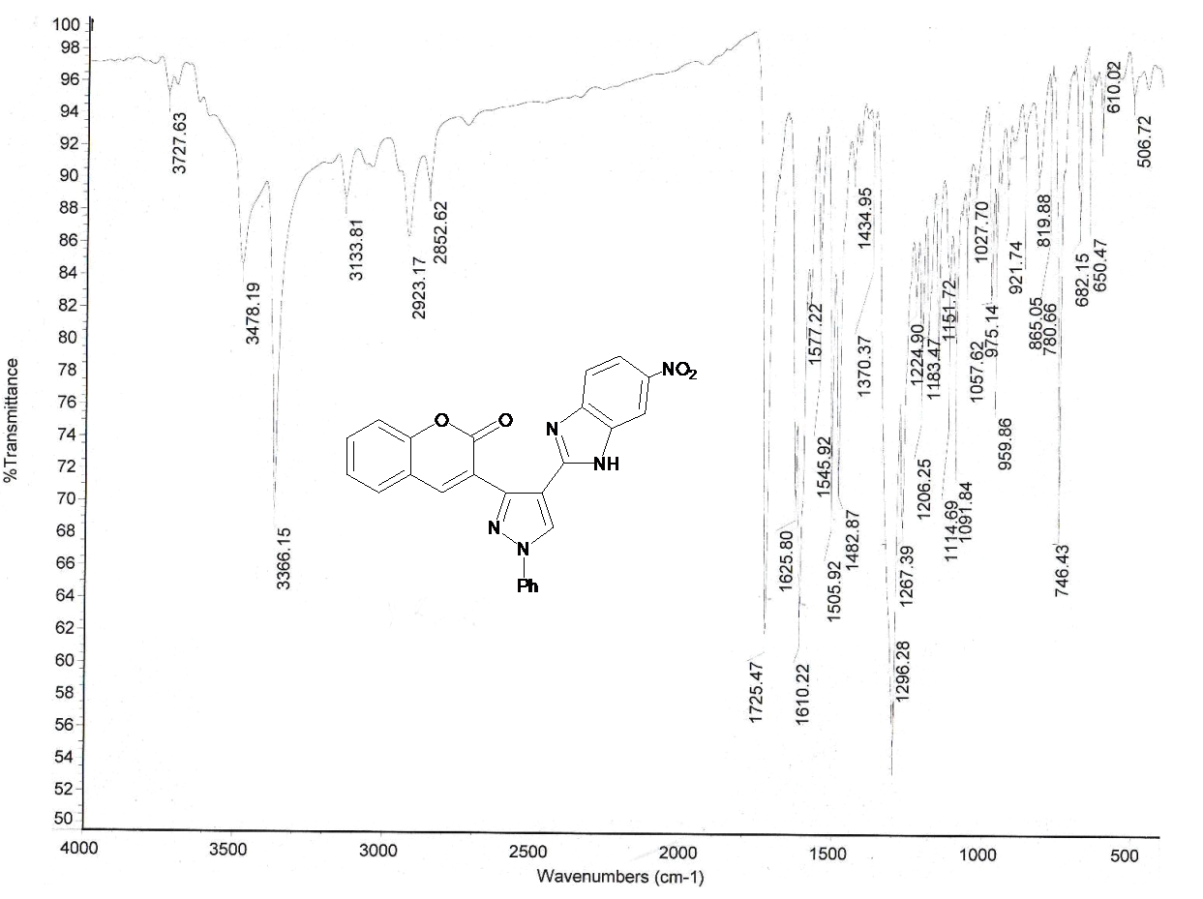
**

**Figure S7. IR Spectrum (KBr) of compound 5d**

**
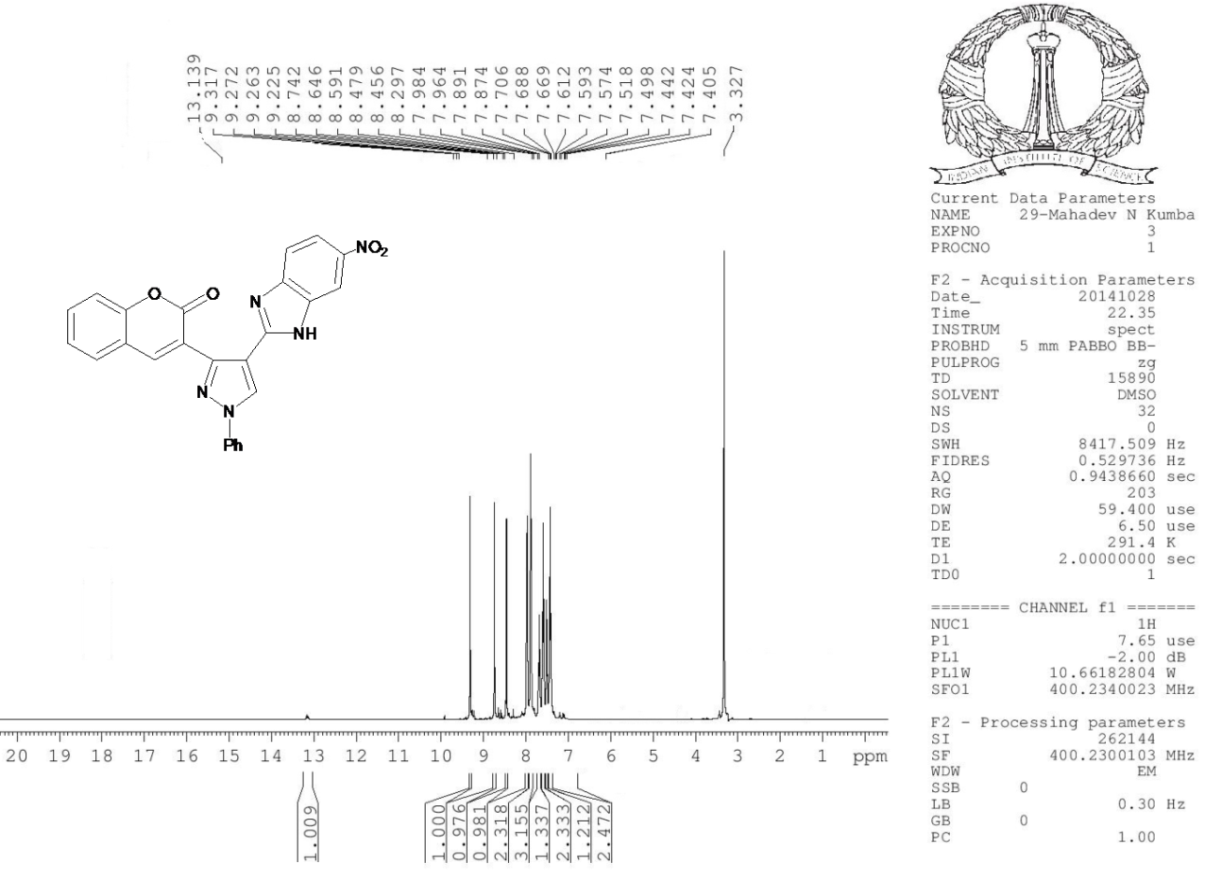
**

**Figure S8. ^1^H NMR Expansion Spectrum (DMSO-d_6_) of Compound 5d**

**
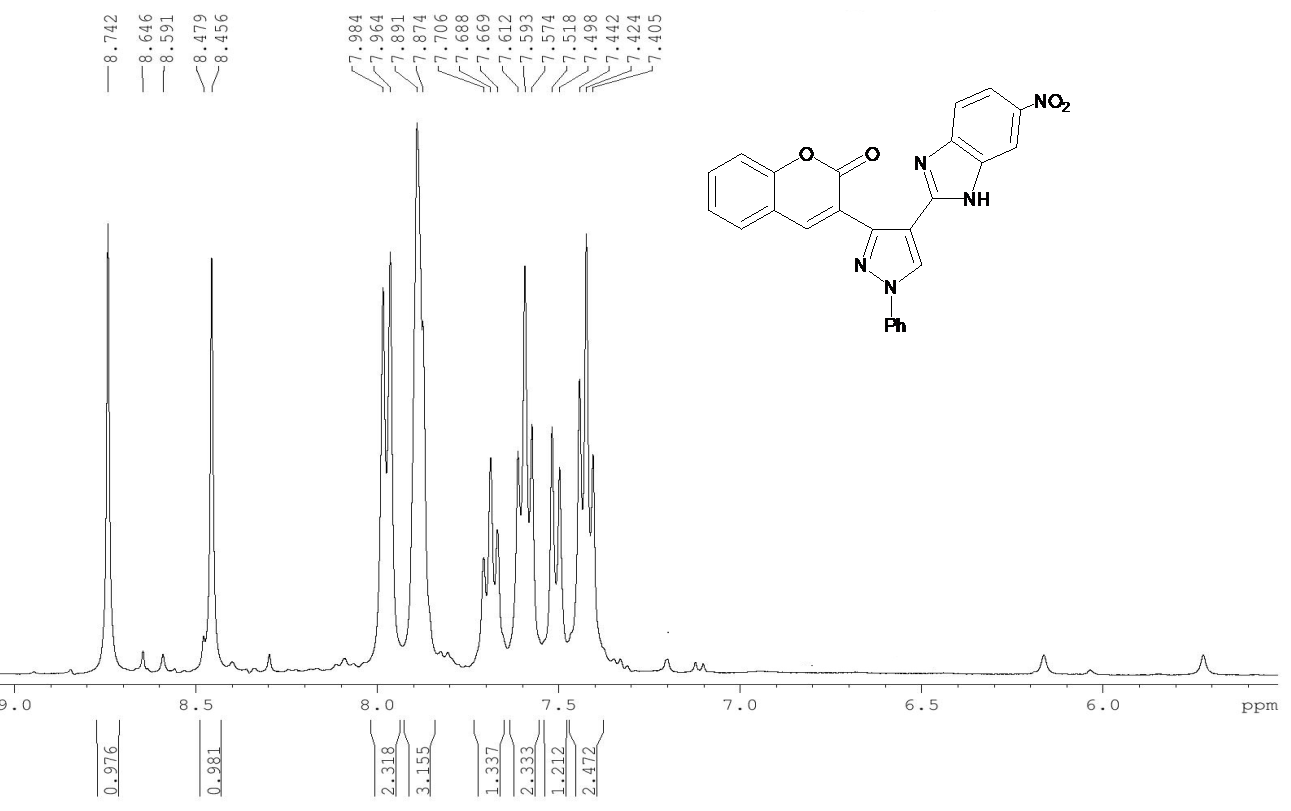
**

**Figure S9. ^1^H NMR spectrum (DMSO-d6) of compound 5d**

**
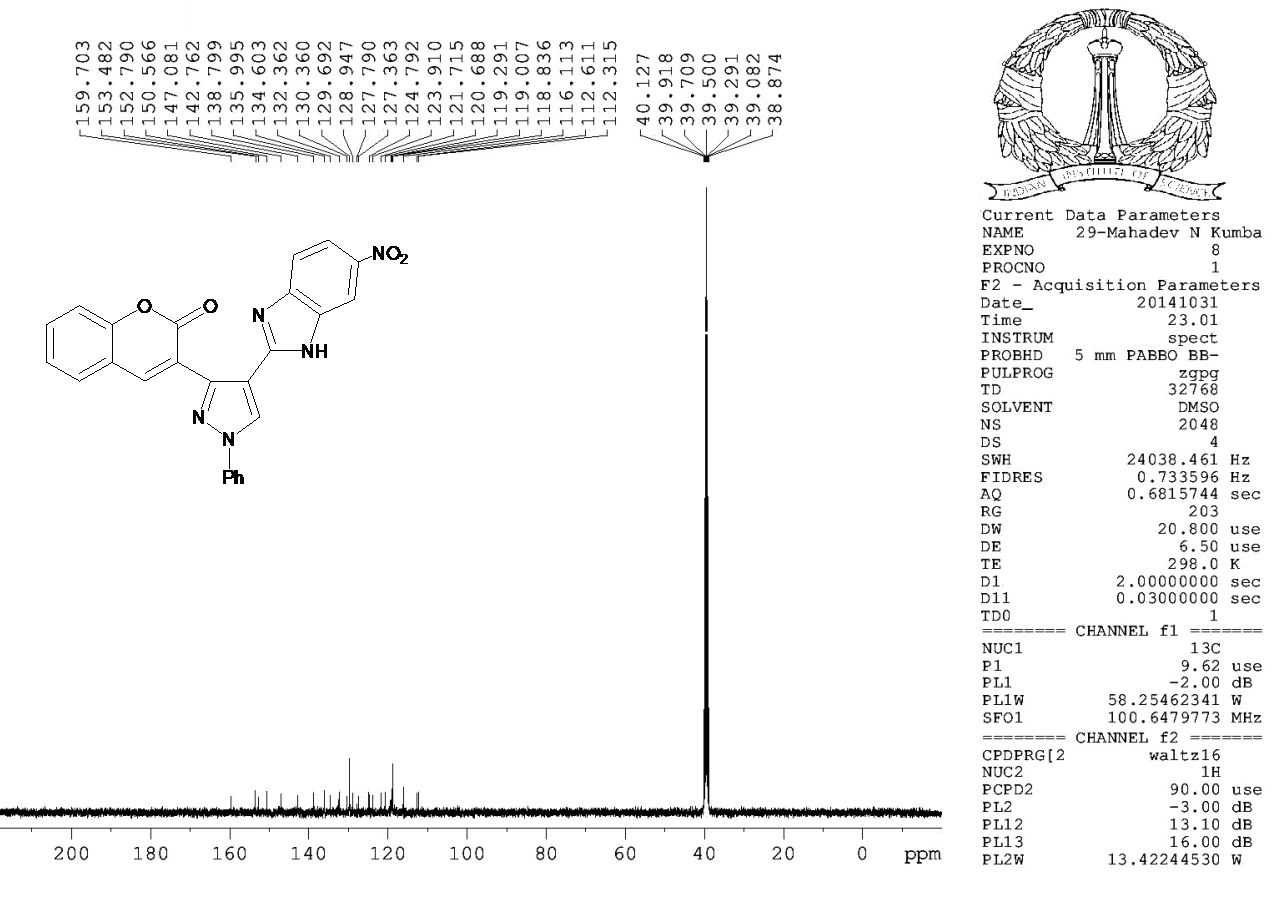
**

**Figure S10. ^13^C NMR Spectrum (DMSO-d6) of Compound 5d**

**
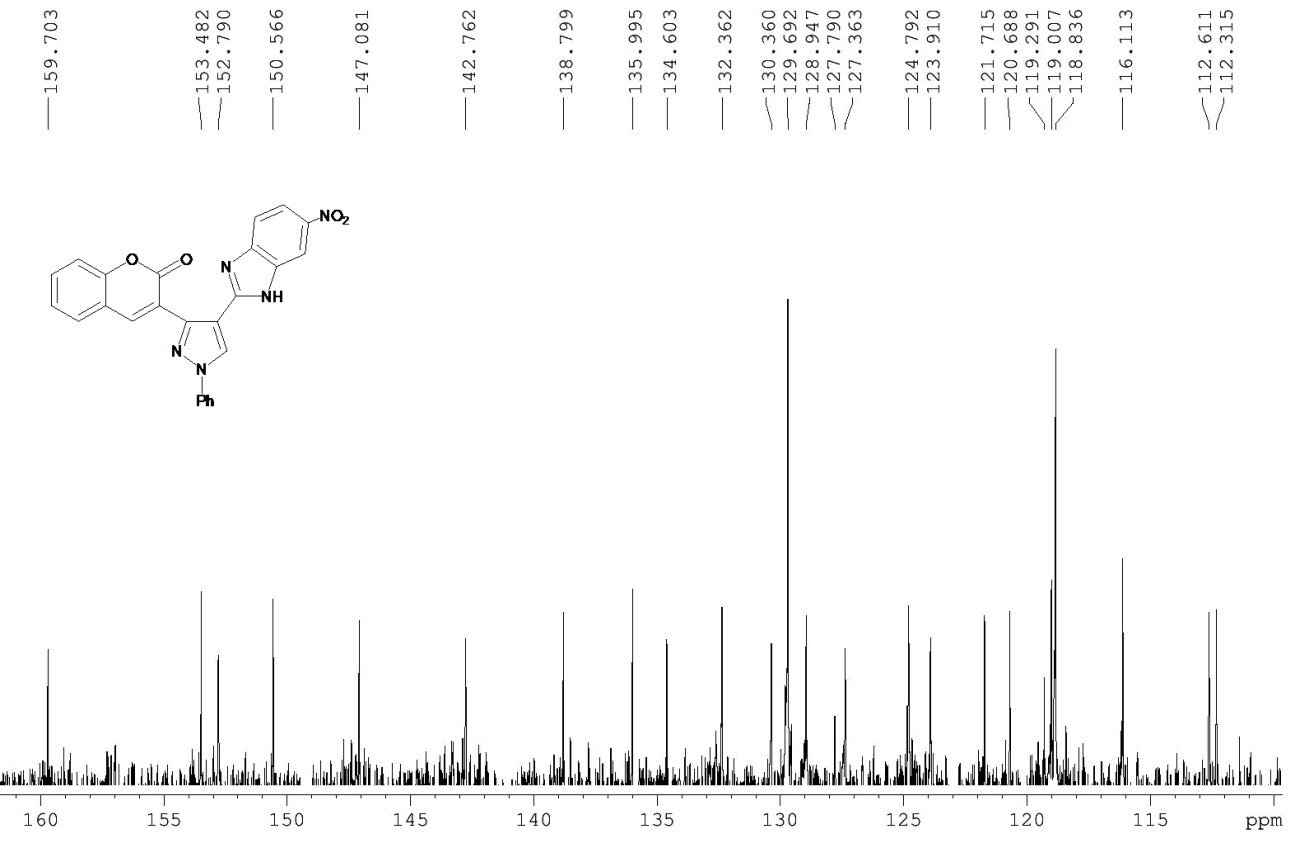
**

**Figure S11. ^13^C NMR Expansion Spectrum (DMSO-d6) of Compound 5d**

**
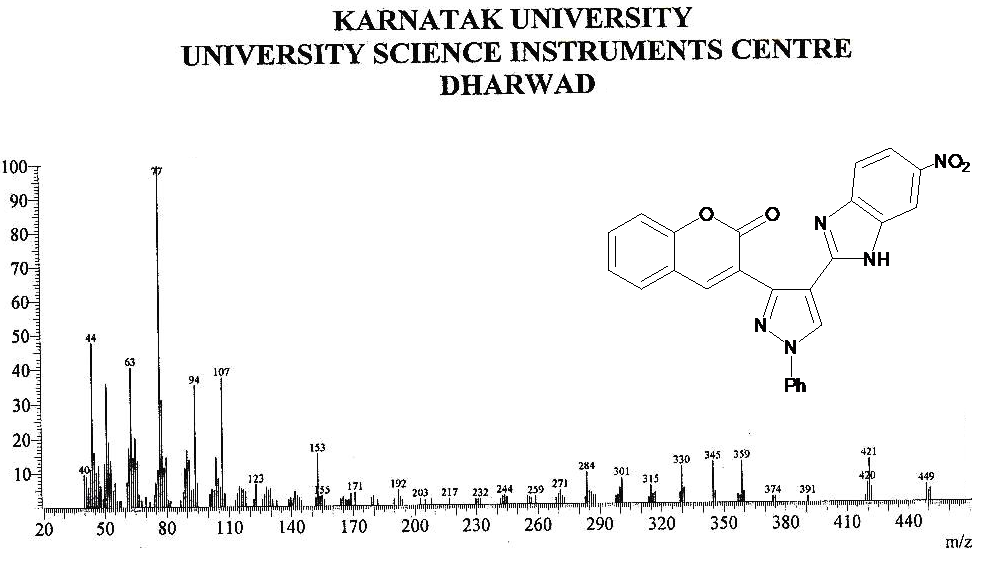
**

**Figure S12. Mass Spectrum of compound 5d**

**
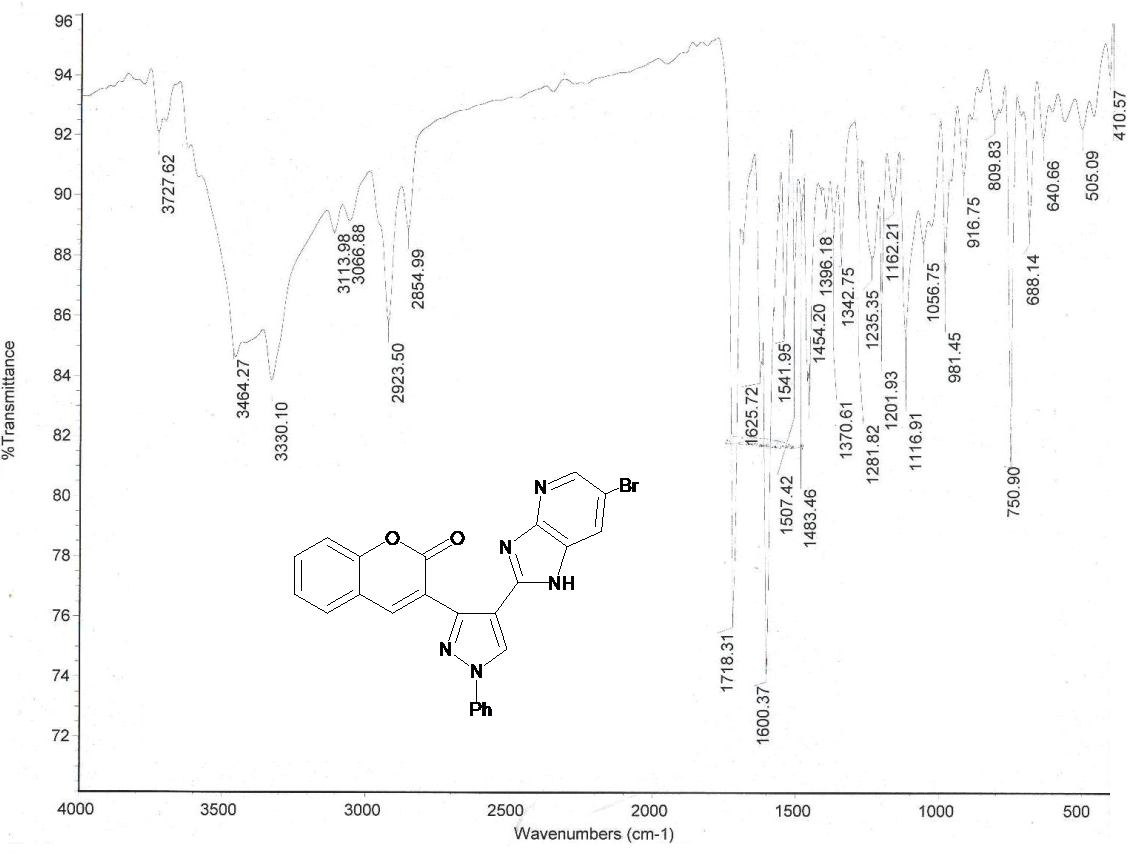
**

**Figure S13. IR Spectra (KBr) of compound 5e**


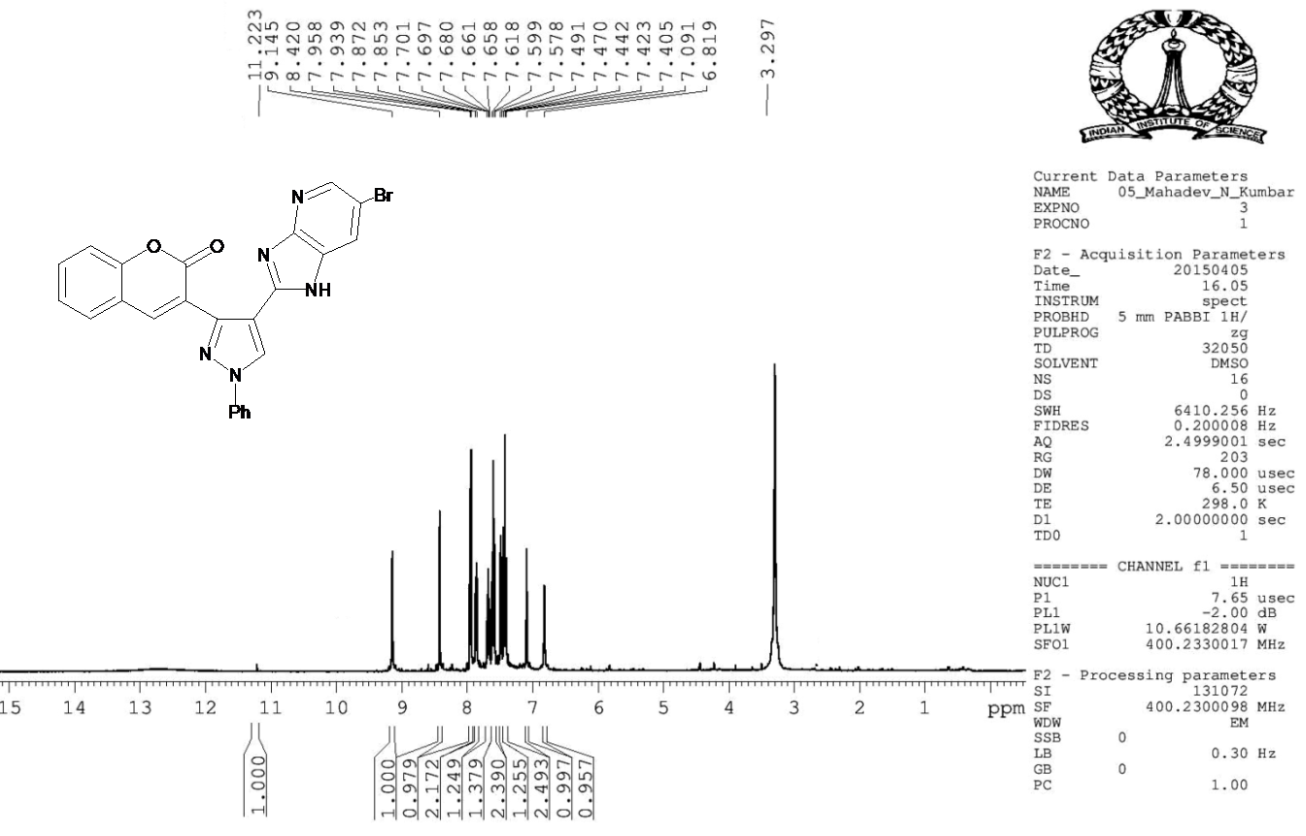


**Figure S14. ^1^H NMR Spectra (DMSO-d6) of Compound 5e**


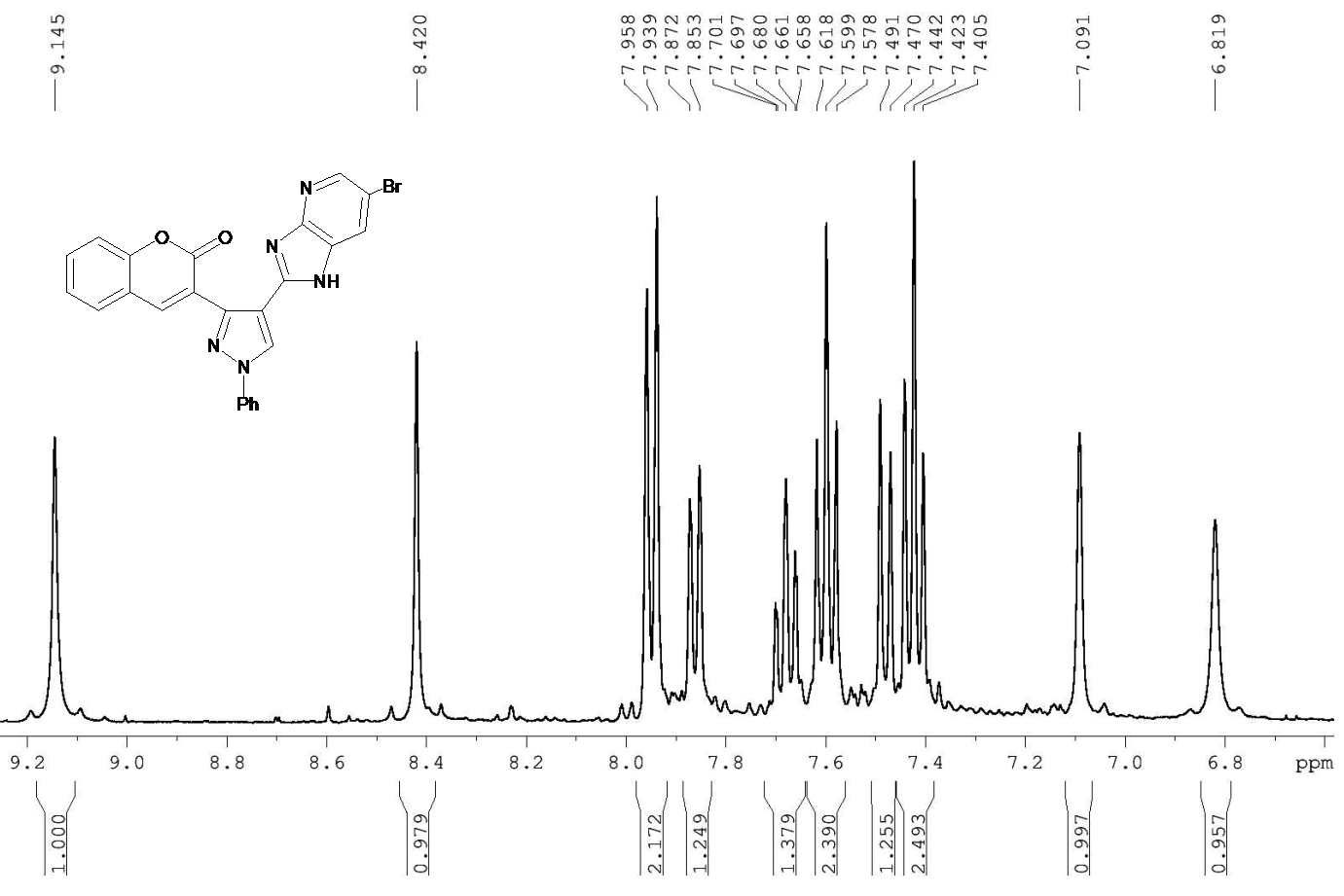


**Figure S15. ^1^H NMR Expansion Spectrum (DMSO-d6) of Compound 5e**


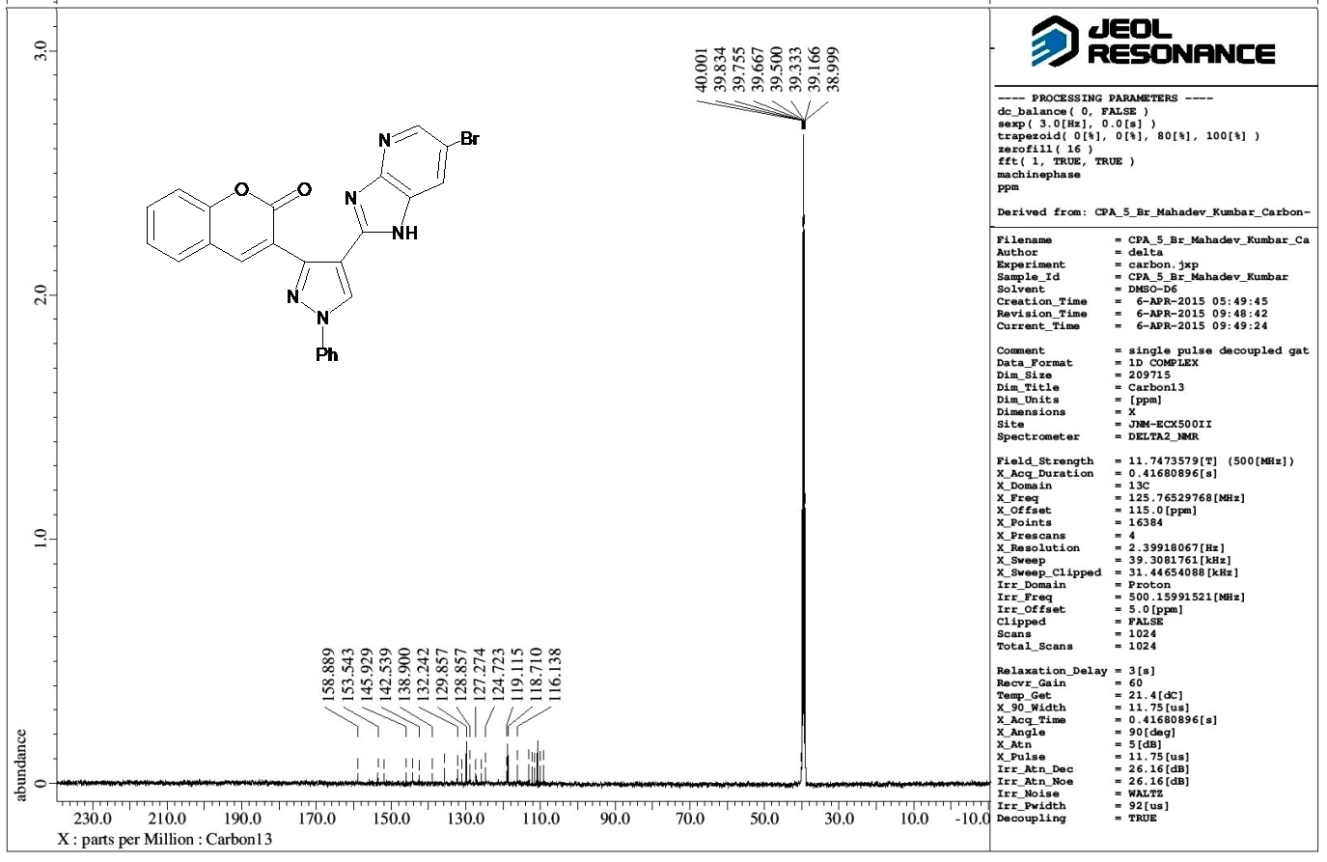


**Figure S16. ^13^C NMR Expansion Spectrum (DMSO-d6) of Compound 5e**


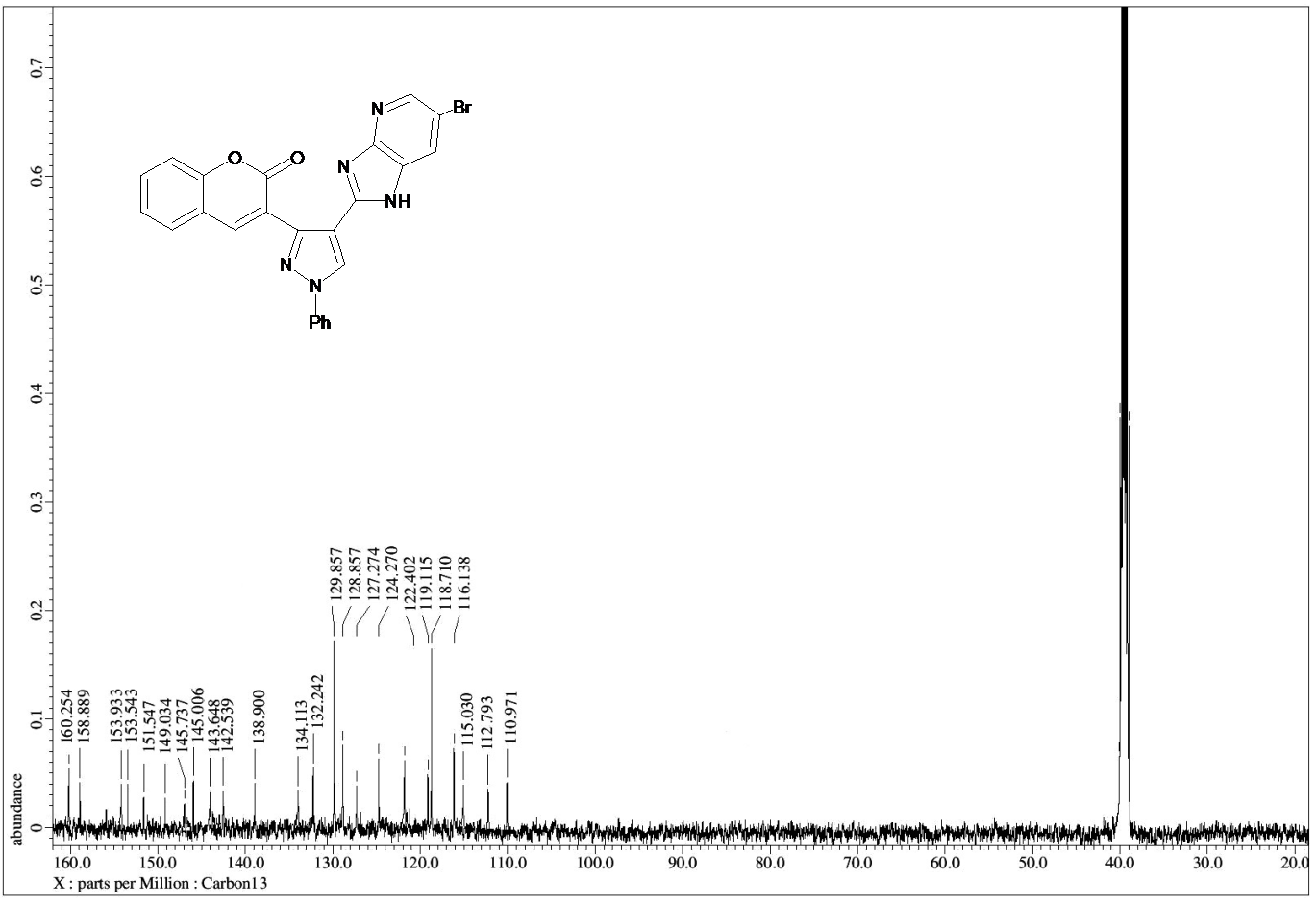


**Figure S17. ^13^C NMR Expansion Spectrum (DMSO-d6) of Compound 5e**


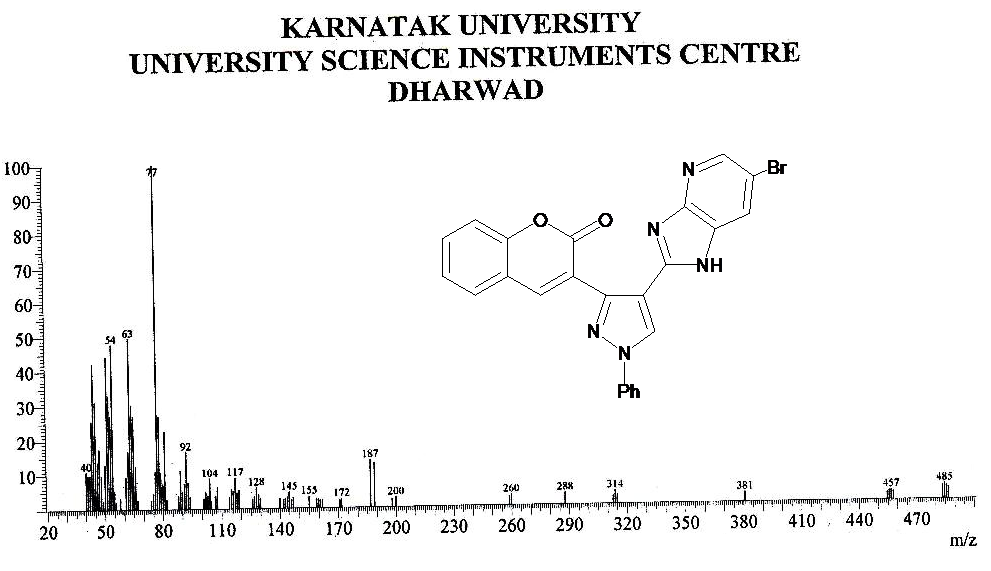


**Figure S18. Mass Spectrum of compound 5e**
